# Impact of exercise to treat postural orthostatic tachycardia syndrome: a systematic review

**DOI:** 10.3389/fneur.2025.1567708

**Published:** 2025-04-24

**Authors:** Melissa M. Cortez, Kayla Aikins, Amy C. Arnold, Jeffrey R. Boris, Todd E. Davenport, Katie Johnson, Hagar S. Kattaya, Laurence Kinsella, Mary M. McFarland, Ryan Pelo, Clayton D. Powers, Kelsi Schiltz, Lauren E. Stiles, Lauren Ziaks, Tae Hwan Chung, Claudia Dal Molin

**Affiliations:** ^1^Department of Neurology, University of Utah, Salt Lake City, UT, United States; ^2^Reno School of Medicine, University of Nevada, Reno, NV, United States; ^3^Department of Neuroscience and Experimental Therapeutics, Pennsylvania State University College of Medicine, Hershey, PA, United States; ^4^Jeffrey R. Boris, MD LLC (clinic), Moylan, PA, United States; ^5^Department of Physical Therapy, School of Health Sciences, University of the Pacific, Stockton, CA, United States; ^6^Workwell Foundation, Santa Rosa, CA, United States; ^7^Orthopedic Center, University of Utah, Salt Lake City, UT, United States; ^8^Department of Neurology, Zaitoun Specialized Hospital, Cairo, Egypt; ^9^Negida Academy, Medical Research Group of Egypt (MRGE), Arlington, MA, United States; ^10^SSM Health Medical Group, Fenton, MO, United States; ^11^Spencer S. Eccles Health Sciences Library, University of Utah, Salt Lake City, UT, United States; ^12^Sports Medicine Department, University of Utah, Salt Lake City, UT, United States; ^13^Kutcher Clinic for Sports Neurology, Park City, UT, United States; ^14^Department of Physical Therapy, University of Utah, Salt Lake City, UT, United States; ^15^Department of Health & Kinesiology/Physical Therapy, University of Utah, Salt Lake City, UT, United States; ^16^Rogue Rehabilitation and Performance, Salt Lake City, UT, United States; ^17^Department of Neurology, Stony Brook University Renaissance School of Medicine, Stony Brook, NY, United States; ^18^Dysautonomia International, East Moriches, NY, United States; ^19^Rehabilitation Services, Park City Hospital, Intermountain Health, Park City, UT, United States; ^20^Department of Physical Medicine and Rehabilitation, Johns Hopkins University, Baltimore, MD, United States; ^21^Department of Orthopedics, University of Maryland School of Medicine, Baltimore, MD, United States

**Keywords:** POTS, exercise rehabilitation program, autonomic, systematic review, synthesis

## Abstract

**Background:**

Postural orthostatic tachycardia syndrome (POTS) is a chronic condition associated with a high symptom burden and decreased quality of life (QOL). Exercise is currently considered to be a first line non-pharmacological treatment for POTS. The purpose of this systematic review was to evaluate the impact of exercise on cardiovascular and patient-centered outcomes in patients with POTS.

**Purpose:**

To evaluate whether exercise benefits patients with POTS by synthesizing data from published clinical studies.

**Methods:**

Electronic databases, including Medline, Embase, CINAHL Complete, Cochrane CENTRAL, and others were searched and results were exported on May 2, 2023. Study inclusion: those that utilized an exercise program as an intervention for POTS and were conducted as experimental or quasi-experimental design. Exclusions: Non-English language papers and opinion-based/theoretical/non-empirical studies/case reports. Data extraction was based on Cochrane Handbook guidance and summarized according to Synthesis Without Meta-analysis (SWiM) guidelines; methodological quality and risk of bias was evaluated using the JBI Critical Appraisal tools. Standardized effects were calculated and summarized based on the direction of effect.

**Results:**

Seven studies included in the final review are described in the data summary and synthesis. Improvements in heart rate were reported across all studies reviewed, while stroke volume and QOL improvements were also found. Notably, not all studies reported on the latter two outcomes. Methodological variability across studies precluded meta-analysis, and risk of bias was considered moderate-high in all but a single study.

**Conclusion:**

While currently available evidence supports exercise as beneficial to QOL and cardiovascular features of POTS, we identified a major need for additional studies assessing the effect of exercise on symptom burden and daily function, including studies that consider patients with specific comorbidities that impact exercise tolerability and/or dosing.

## Introduction

Postural orthostatic tachycardia syndrome (POTS) is a chronic disorder of the autonomic nervous system that predominately affects young people (1). Although the prevalence of POTS is unknown, prior to the COVID pandemic it was estimated to affect up to 3 million individuals in the US (2) – and many more cases have been diagnosed following COVID-19 infections (3). Current consensus criteria for the diagnosis of POTS in adults requires symptoms of orthostatic intolerance for at least three months, along with a sustained increase in heart rate (HR) of at least 30 beats per minute within 10 min of upright position (40 beats per minute in patients age 12–19 years) (4–6). Current criteria also stipulate the exclusion of other potential causes for postural tachycardia and related symptoms, such as orthostatic hypotension, dehydration, medication effects, endocrine dysfunction, and deconditioning resulting from prolonged bedrest.

In addition to symptoms of orthostatic intolerance and postural tachycardia, patients with POTS suffer from multisystemic symptoms, including fatigue and exercise intolerance (7, 8). Such symptoms often critically impact quality of life (QOL) and daily function, and an estimated 70% of adults with POTS have lost income due to their symptoms (9). Despite this high prevalence and rate of disability, there are currently no FDA-approved medications for POTS. While medications are often used, structured therapeutic exercise – generally beginning with aerobic and strength training using recumbent positioning, gradually building toward longer durations and a more upright posture – remains a cornerstone of current consensus-driven POTS management.^5,6^ However, the literature lacks a critical summary of the efficacy and tolerability of current exercise protocols, including whether common comorbidities impact treatment responses.

### Objective

The aim of this systematic review is to summarize the literature evaluating the impact of exercise as a therapy for patients with POTS, inclusive of those with or without comorbid conditions that may impact exercise prescriptions, and to provide a synthesis of findings from such studies, along with appraisal of their quality and risk of bias, while identifying gaps that warrant further investigation.

## Methods

This systematic review was conducted with guidance from the *Cochrane Handbook for Systematic Reviews of Interventions* and adheres to the PRISMA and Synthesis Without Meta-analysis (SWiM) reporting guidelines (10, 11). See Supplementary material for detailed reports of review search criteria, as well as included and excluded articles. The *a priori* protocol is registered on PROSPERO CRD42023421863 (12).

### Inclusion criteria

Studies were included if they utilized a structured exercise therapy program as an intervention to treat POTS and were conducted as experimental (randomized clinical trials; RCTs) or quasi-experimental design, including non-randomized clinical trials, cohort studies (retrospective/prospective), and cross-sectional studies. Persons of any age or sex with a diagnosis of POTS with or without included comorbidities were included in the studies. POTS diagnosis was based on laboratory, clinical, or reported history. Clinically reported or diagnosed comorbidities included Ehlers-Danlos syndrome (EDS), hypermobility spectrum disorder, autoimmune disease, post-acute infection syndromes (e.g., Long-COVID), concussion, mast cell disorders (e.g., mastocytosis, mast cell activation syndrome [MCAS]), and disorders characterized by orthostatic intolerance and/or post-exertional malaise (PEM), including myalgic encephalomyelitis/chronic fatigue syndrome (ME/CFS). Control participants were not required.

The primary outcomes were change in HR and/or stroke volume (SV). Change in HR was defined as a change in supine HR before and after exercise training, a change in upright HR before and after exercise training, or a change in orthostatic HR increment (the difference between upright and standing HR) before and after exercise training. Similarly, change in SV was defined as either a change in supine SV before and after exercise training or a change in upright SV before and after exercise training. The secondary outcome of interest was QOL. Additional secondary outcomes of interest, including symptom burden, daily function, and disability were included in our registered protocol; however, these were not included in the final analysis, as fewer than two studies reported these measures.

### Exclusion criteria

Non-English language papers were excluded at full text review, as were opinion-based, theoretical, and/or non-empirical studies, case reports, case series, reviews, and guidelines.

### Information sources and search strategy

The search for this review was designed to find evidence for the research question, “In patients with POTS of any age or sex with or without selected comorbidities, do structured exercise interventions affect outcomes of QOL, disability, symptom burden, stroke volume and/or heart rate?” An information specialist [MMM] developed the search strategy using a combination of keywords and database subject headings, sentinel studies, and team feedback, which were deployed in a primary database (Medline) and later translated to the remaining selected databases. Search terms included: POTS, postural orthostatic syndrome, postural orthostatic tachycardia syndrome, postural tachycardia syndrome, tachycardia, orthostatic intolerance, exercise, exertion, exercise intolerance, and exercise therapy. The search strategy was then peer reviewed by a library colleague according to PRESS guidelines to ensure a comprehensive, balanced search, providing our evidence base (13). See Supplementary material 1 for detailed search strategies.

Electronic databases included Medline (Ovid) 1946–2023, Embase (Elsevier) 1974–2023, CINAHL Complete (Ebscohost) 1937–2023, Cochrane CENTRAL (Wiley) 1898–2023, APA PsycINFO (Ebscohost) 1872–2023, Psychology and Behavioral Sciences Collection (Ebscohost) dates vary by title, SportDiscus (Ebscohost) 1800–2023, Scopus (Elsevier) 1970–2023 and Web of Science Core Collection (Clarivate) 1900–2023. No date nor methodology filters were applied to databases, although conference abstracts were excluded from Embase, Scopus, and Web of Science. Grey literature searches were not conducted. Searches were executed and exported on May 2, 2023. EndNote, version X9 (Clarivate Analytics) was used to manage citations and remove duplicates from search results; Covidence (Veritas Health Innovation) provided an additional second pass for removing duplicates. References of included studies were checked for relevancy. No other methods were utilized to find studies.

### Selection process

Two reviewers [CD, KA] independently screened all titles and abstracts, followed by independent review of full text articles, to ensure inclusion and exclusion criteria were met. When conflicts could not be resolved through discussion, a third reviewer [MMC] cast the deciding vote. Covidence (Veritas Health Innovation) an online systematic reviewing platform was used to screen and track studies.

### Data collection process and data availability

Two reviewers from a pool of three [KA, CD, and MMC] independently extracted the following data from included studies using Microsoft Excel, version 2019: study characteristics (participant inclusion and exclusion criteria, exercise therapy, and outcomes measured), participant characteristics (age, sex, comorbidities), methods (blinding, randomization, modality of exercise therapy, duration, intensity, targeted area, frequency, and control method), and outcomes (physiological and self-reported outcomes) were recorded. Data were reviewed and confirmed by a further reviewer [HSK] prior to synthesis. Where incomplete or unclear elements of the data were encountered, clarification and/or additional data was requested from authors. Data supporting this review are available upon reasonable request to the corresponding author.

### Synthesis methods and effect estimates

Due to observed heterogeneity across reports in terms of outcomes, exercise interventions, time points of assessment, and the overall small sample size for any given outcome, a systematic review with meta-analysis was determined to be inappropriate and thus was not included in the review protocol. Instead, a systematic review and synthesis without meta-analysis (SWiM) was conducted (11). Studies found to be reporting, on overlapping, experimental datasets were merged for results reporting, and synthesis, according to Cochrane Handbook guidance. To synthesize results across reports, we used vote counting based on the direction of effect for each outcome, according to published methods (14). In all cases, effect of the exercise intervention on the designated outcome was based on the reported pre−/post- intervention data (i.e., within individual comparisons). Standardized effects were calculated for each outcome that was reported in two or more studies (reported as standardized mean differences and 95% confidence intervals; CIs). Each effect estimate was then categorized as a positive health impact (i.e., a clinically beneficial change), or not, based on the direction of effect. Where there were multiple potential reporting methods across studies for a particular outcome domain (e.g., where treatment effect on HR was reported as supine, upright, and/or the supine-upright delta, all possible options included within the “HR outcome domain”), the overall direction of effect for the domain was determined by synthesizing the directions of effect for all outcomes within a domain by calculating the proportion of effects for positive and negative health impact, respectively. Review Manager (RevMan) software version 5.4.1 (Copenhagen: The Cochrane Collaboration, 2020) was used to calculate standardized effect sizes for all outcomes and to summarize the overall direction of treatment effect. GraphPad^1^ was used to calculate the two-tailed *p*-value for each outcome domain.

### Study risk of bias and quality assessment

JBI critical appraisal tools,^2^ were used to provide a quality assessment for each included report. The appropriate appraisal tool was selected for non-randomized studies (15) and RCTs (16). These tools were used to assess key aspects of study design for each included report, as well as evaluation of internal validity and generalizability based on reporting within each article. As per our registered protocol (12), our initial plan was to also apply risk of bias evaluations, via ROB 2 (17) for RCT and ROBINS-I (18) for non-randomized studies, and to provide GRADE ratings (19). However, after our initial application of the JBI tools, we found the JBI risk of bias appraisals to be adequate such that the additional use of ROBINS and ROB-2, as stated in the published protocol, would have been redundant. The latter decision was based on a consensus among our writing group members relative to the overall quality and risk of bias across studies where it was believed that the data and evidence were insufficient to support a formal GRADE recommendation (discussed further below in Results and Discussion).

A single investigator determined the appropriate critical appraisal tool to use for each included study [TED]. Two additional investigators then used the tools independently to rate risk of bias for each article [LZ and CDP]. Discrepancies were resolved by the first investigator [TED]. Quality was downgraded based on methodological limitations identified using each evidence appraisal tool. Additional downgrading was assigned to studies that had methodological limitations that were not a part of each tool but were determined to be important based on expert opinion. Specifically, failure to apply a consensus-based case definition for POTS resulted in an additional downgrade. The quality of the evidence was then summarized as “low risk of bias,” “some concerns,” or “high risk of bias,” according to JBI score and any relevant additional downgrading.

## Results

### Study selection

**Figure 1:**
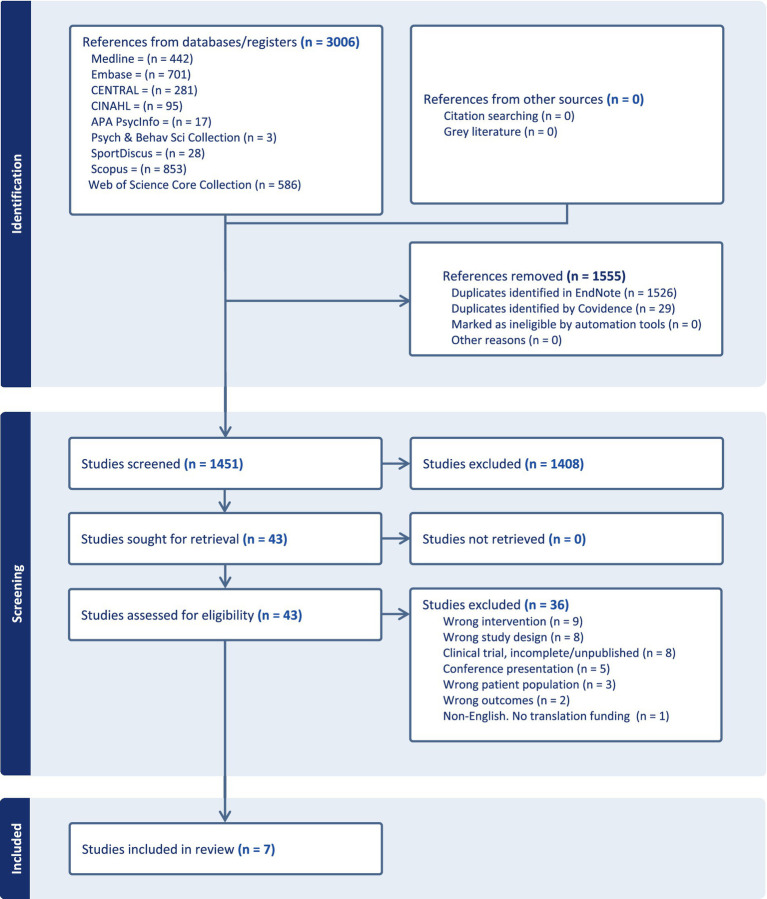
PRISMA diagram.

After duplicates were removed, 1,451 published studies were screened; of these, 43 were selected for full text review, resulting in 8 total studies included in the qualitative assessment. Of the 8 final studies identified, the Fu et al. (20) and Fu et al. (21) reports were merged for summary and synthesis according to Cochrane Handbook guidance, based on their report of the same study cohort and outcomes, yielding an effective total of 7 reports included in the review. The flow diagram of the results and screening process was shown in Figure 1.

### Study characteristics

**Figure 2:**
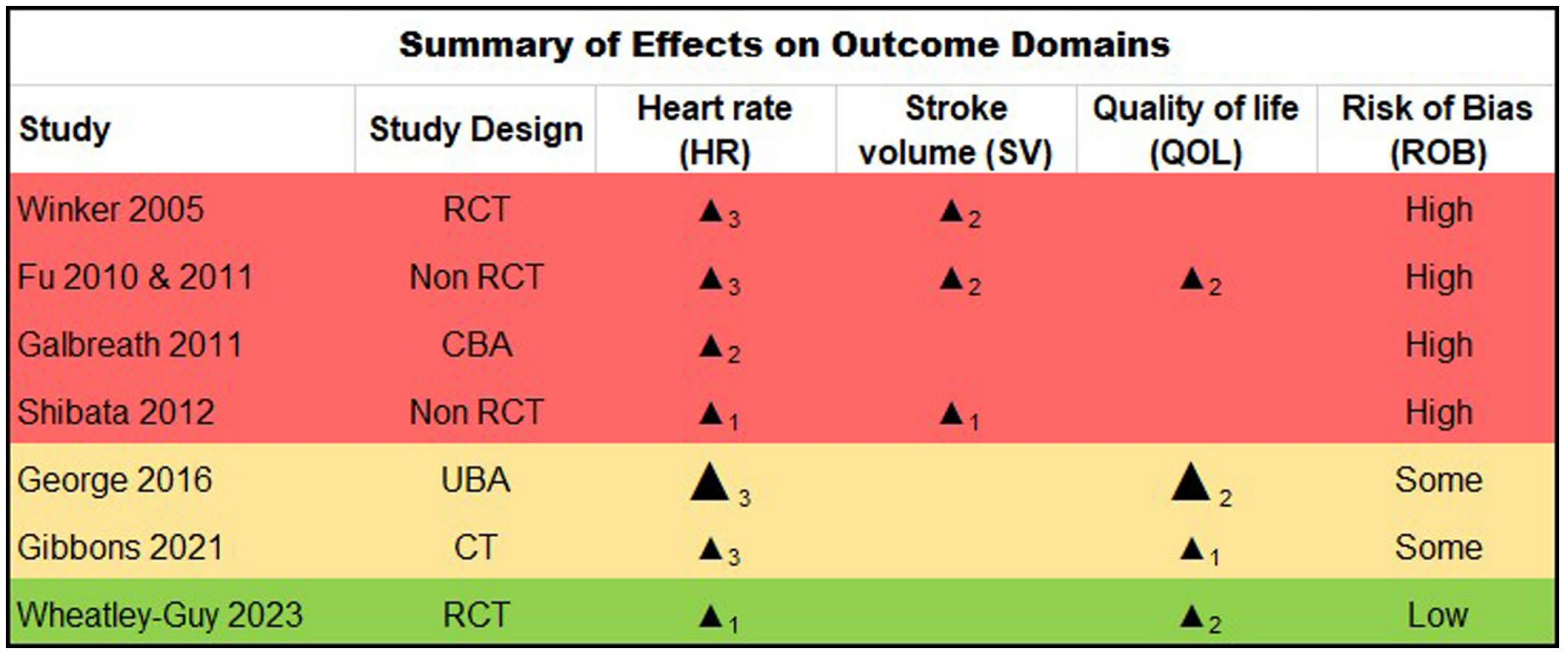
Study design. RCT: Randomised Controlled Trial; CBA: Controlled Before & After study, UBA: Uncontrolled Before & After study, CT: clinical trial. Summarized Direction of Effect (Benefit on Outcome): upward arrow = positive health impact, downward arrow = negative health impact. Sample size: Final sample size (individuals) in intervention group, Large arrow >50, small arrow <50. Subscript numbers: Number of outcomes within each outcome domain (HR, SV, QOL) that were reported within each study. (e.g., for the HR domain, subscript 3 denotes that all three possible definitions of HR change were reported within the given study; for the QOL domain, subscript 2 denotes two-domain QOL instrument reported for the given study). Study quality: denoted by row color, green = low risk of bias, amber = some concerns, red = high risk of bias.

The characteristics of the 7 eligible studies are summarized in Figure 2, including study design and designated outcomes, along with synthesis based on direction of effect and risk of bias.

### Risk of bias assessment

Quality of evidence and risk of bias findings are shown in Figure 2. Five reports were rated at a high risk for bias (20–24), two at moderate risk (25, 26) and one at low risk (27). In general, methodological quality was moderate across the majority of randomized and non-randomized studies reviewed (63.8 and 78.9% respectively). The key limitations noted across reports included sample size factors, limited generalizability, and the nature of multiple dependent variable measurements. Four small studies appeared to report on the same participants (*n* < 20) (20–23), albeit using slightly differing analytic approaches and outcomes. Thus, data from this subset of participants are effectively over-represented in the results. Several studies reported low enrollment relative to the number screened (27), calling into question their generalizability, and dropout/adherence rates were generally high or inconsistently reported.

**Figure 3:**
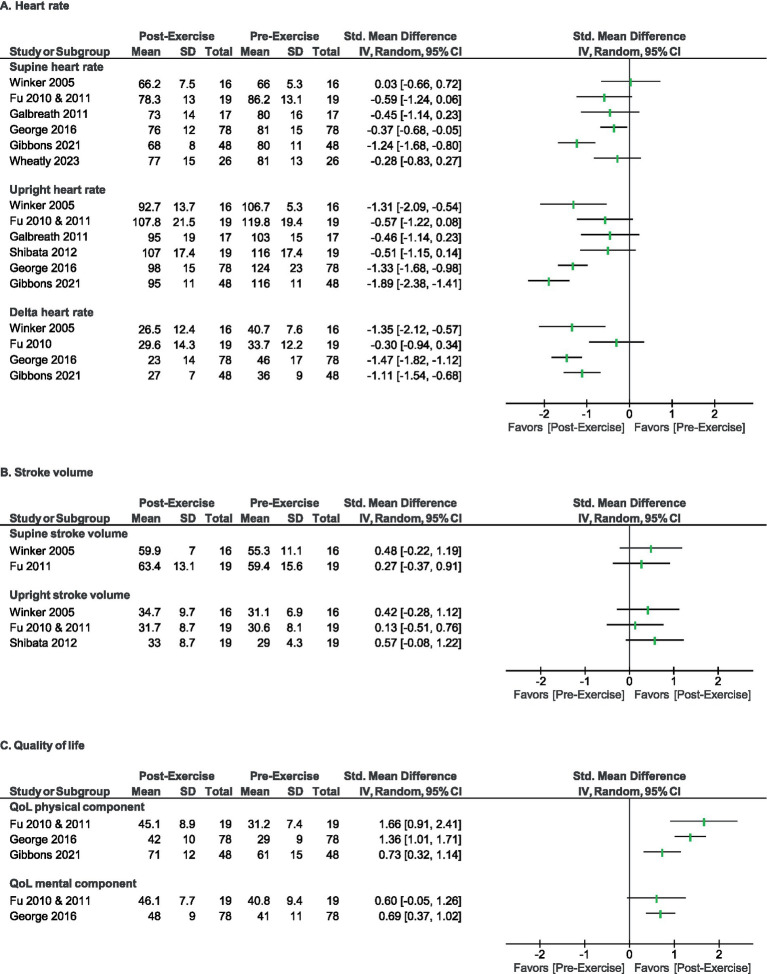
Direction of effect for primary outcomes for heart rate **(A)**, stroke volume **(B)**, and quality of life **(C)**. Forest plots summarizing direction of effect for each outcome domain within reviewed studies.

Although exercise positively affected the designated outcomes in the majority of studies (Figure 2), the magnitude of these changes were often modest (Figure 3), and were not always comparable to a matched control, calling into question their degree of clinical meaningfulness and/or whether the exercise intervention caused a unique effect in the participants with POTS compared to exercise effects in deconditioned, chronically ill, or otherwise healthy people. Finally, per-protocol and last observation carried forward type analysis approaches were common across reports (rather than intention to treat analyses), which may bias summary interpretation as well.

### Results of individual studies

Narrative summaries of the 7 included studies are presented here, listed in chronological order. Of note, four of the listed studies (20–23) were derived from the same study participants (denoted with an asterisk), with differing approach to data analysis and the derived conclusions. Thus, for purposes of descriptive summary, details of each report relative to their intended analyses are included below for reference:

Winker et al. (24) completed a randomized controlled trial consisting of 31 young males in active military service with the Austrian army diagnosed with idiopathic orthostatic intolerance defined as an increase of at least 30 bpm in heart rate after a tilt table test and plasma norepinephrine concentrations >600 pg./mL after 30 min upright during the tilt-table examination. Participants were randomly allocated to two groups: “training” (*n* = 16) or “non-exercise control” (*n* = 11; initially 15 with 4 lost to follow-up). Outcomes included HR and BP during tilt table testing and a symptom questionnaire, collected at an initial visit and 3-months. The exercise training group completed a 3-month program consisting of a jogging progression with three 1-month stages, incrementally increasing in session duration. The study found that the training group had fewer individuals that experienced a 30 bpm or more increase in heart rate during a 30-min tilt test (10 of 16; 63%), compared to the control group (where 10 of 11, 91% retained their abnormal HR response to orthostatic challenge). The training group also showed an improvement in the average orthostatic symptom score based on an occupational orthostatic intolerance assessment questionnaire (28), whereas the control group did not.

Fu et al. (21)* and Fu et al. (20)* reported on a double-blind drug (propranolol) trial followed by a non-randomized exercise run-on phase consisting of 25 subjects meeting HR criteria for POTS, without EDS, and 15 healthy control subjects. The study intervention included a 4-week double blinded drug trial (propranolol 80 mg or placebo), followed by 3 months of exercise training. The 3 month “personalized” exercise training program utilized a modified Astrand-Saltin incremental treadmill protocol to determine individual HR zones of intensity; subjects performed a progressive, phased exercise plan of increasing intensity and orthostatic load (body position). Subjects were encouraged to gradually increase their dietary salt intake to 6–8 grams/day and water intake to 3–4 liters/day. Participants, conservative measures, and exercise intervention were the same between this study, and 3 others identified by our search criteria (20–23). Subjective outcomes reported by Fu et al. (21) included: SF-36 and social functioning score; physiologic outcomes included HR, SV, cardiac output (CO), total peripheral resistance (TPR), blood pressure (BP), blood volume, plasma volume, peak oxygen uptake (aka, VO_2_ max), left ventricular mass and end diastolic volume, and supine hematocrit; assessments were performed off medications that could affect the autonomic nervous system. Results for 19 of 25 participants were included in the final analysis published by Fu et al. (21). Patient-reported QOL improved after exercise training but not after propranolol treatment. After training, VO_2_ max, BV, PV, and left ventricular mass increased, supine and standing HR decreased, and the majority no longer met HR criteria for POTS. Fu et al. (20) reported on additional physiologic outcomes, including orthostatic HR, BP, and blood samples (catecholamines, plasma renin, and aldosterone); QOL (SF-36) was also reported. In this report, by the end of the training phase, subjects were reported to have increased weekly training volume and tolerated more upright body positions. QOL improved after training but not after propranolol treatment; both propranolol and training lowered standing HR. Aldosterone:renin ratios were modestly increased after training, while plasma catecholamines were not altered by either intervention. Training appeared to attenuate plasma renin activity with preserved aldosterone during prolonged standing, so that the aldosterone:renin ratio increased.

Galbreath et al. (22)* conducted a prospective case–control study, reported as a follow-on to a RCT (21) of propranolol comparing pre−/post- exercise intervention results, consisting of 17 participants with POTS >6 months. Subjects were compared against 17 healthy, sedentary volunteers. The 3-month exercise program utilized an incremental approach to increasing intensity and orthostatic load (body position), accompanied by addition of salt and hydration measures, as described in Fu et al. (21). Participants, conservative measures, and exercise intervention were the same between this study, and 3 others (20–23). Arterial-cardiac baroreflex function, along with HR and BP were evaluated in the supine position and at 60 degrees head-up tilt before and after training. Supine and upright HR were significantly greater in POTS participants than controls at baseline. Exercise training decreased upright HR and increased R-R interval variability in the POTS group. The majority (10 of 17; 59%) of experimental group participants no longer met the POTS heart rate criteria.

Shibata et al. (23)* reported on a non-randomized, prospective case–control study, also separately reported, as a follow-on to a RCT of propranolol (21); this study initially enrolled 25 participants with POTS (results reported for 19 POTS participants that completed the intervention; 24% drop-out reported). The POTS group was compared against 10 age-matched healthy, but otherwise sedentary individuals. The 3-month exercise program utilized an incremental approach to increasing intensity and orthostatic load (body position), accompanied by addition of salt and hydration measures, as described in Fu et al. (21). Participants and exercise intervention were otherwise the same between this study and 3 others (20–23). Physiologic assessment were performed in the upright position at baseline and after the exercise training program, including CO, SV, BP, mean arterial pressure (MAP), TPR, maximal oxygen uptake ( $\dot{V}$ O_2_max), including recovery from exercise (calculated from peak exercise to minute 2 of recovery). Results demonstrated that at baseline, a lower SV was associated with a higher HR in POTS at any given oxygen uptake ( $\dot{V}$ O_2_) during exercise, while CO remained normal. $\dot{V}$ O_2_max was lower in POTS than healthy sedentary controls. After the training program, the POTS group had a relatively decreased HR at any given $\dot{V}$ O_2_, associated with an increased SV, without changes in CO. $\dot{V}$ O_2_max increased due to increased peak SV and was proportional to TPR. HR recovery from an acute bout of exercise was faster after training than before training.

George et al. (26) completed a non-randomized clinical trial evaluating pre−/post-exercise responses, evaluating 251 subjects diagnosed with POTS by their local physician. The 3-month self-administered exercise program, provided as written recommendations to the patient’s local physician, included mild to moderate intensity endurance training progressing from semi-recumbent to upright, 3–5 times/week for 30–45 min/session, plus strength training. Participants were also instructed to increase salt to 7–10 grams/day and water to 3 liters/day intake, and to increase the head of the bed 4–6 inches while sleeping. Participants were able to continue existing medications or begin new medications or other treatments during the trial. Physiologic outcomes measured at baseline and at the completion of a 3-month program included HR and BP during 10-minute stand testing. Some patients took longer than 3 months to complete the program. Subjective secondary outcome included QOL measured by the SF-36. The study reported 59% (148 of 251) dropout. Of those who completed the study, the authors reported that 71% no longer met the heart rate criteria for POTS on follow-up 10-min stand, where the increment in HR (supine to standing) markedly decreased, while patient-reported QOL showed a statistically significant improvement over baseline.

Gibbons et al. (25) conducted a pragmatic clinical trial consisting of 77 subjects diagnosed with POTS who were offered the opportunity to participate in a 6-month unsupervised exercise program. Of note, the investigators screened 230 patients with postural tachycardia, excluding 141 due to medication use (antidepressants, methylphenidate, antihypertensives, opiates for pain, etc.) and 12 due to medical conditions known to cause tachycardia, specifically diabetes and hyperthyroidism. Some patients in the study had co-morbid EDS. Individuals who opted out of exercise were designated as “controls,” and were provided routine clinical care recommendations regarding fluid and salt intake. Of the 77 patients invited to participate in the study, 48 (62%) elected to participate in the exercise group; 19 participants who opted out of exercise and 10 who did not follow-up (total of 29 participants), were treated as a control group for analysis. The 6-month exercise program consisted of recumbent to upright exercise 6 days/week, with progression in session duration from 10 to 45 minutes over time. Physiologic outcomes included HR and BP during tilt table testing. Secondary outcomes included the EuroQol Perceived QOL Scale, collected within 6 months of initiating the exercise protocol and within 2 months of exercise protocol completion. The study reported 10% dropout (43 of 48 completed the exercise program). After 6 months, 23% of individuals in the exercise group met HR criteria for POTS, compared with 93% in the control group. A greater improvement in the EuroQol perceived QOL scale score was detected in the exercise compared to the control group. Notably, 10 of the 29 participants included in the control group did not have follow up data available, thus the investigators carried forward their first visit data (last observation) to calculate the 6-month follow-up data used for analysis.

Wheatley-Guy et al. (27) documented the results of a randomized controlled trial evaluating 60 individuals with POTS. Inclusion in the study allowed designated comorbidities of: migraine/headaches, MCAS, asthma, fatigue, pain, irritable bowel syndrome (IBS), anemia, Sjögren’s. Participants were randomized to either exercise training (*n* = 31) or standard of care (*n* = 29) groups. Individuals with hypermobile EDS or hypermobility spectrum disorder were intentionally stratified (randomly) into groups to ensure equal amounts of participants with hypermobile EDS between groups. The exercise training group received an exercise consultation and 8 semi-supervised in-person or virtual exercise sessions, versus the standard of care group, who followed recommendations of primary neurologist or cardiologist for symptom management (salt intake, water intake, physical therapy, medications, and aerobic exercise). Outcomes included: COMPASS-31, 10-minute stand test, and cardiopulmonary exercise test performed at baseline and after the 12-week intervention. Of the initial 60 recruited, 11 withdrew; 5 of the initial 31 in the exercise group, and 6 in the standard of care group. The study found that the exercise training group demonstrated a greater improvement in $\dot{V}$ O_2_max, improved tolerance for peak workload, and more often had a delayed orthostatic symptom onset with exercise than the standard of care group. Individuals in the exercise training group had lower COMPASS-31 orthostatic intolerance domain scores after the intervention, though total COMPASS-31 scores did not differ.

### Results of data synthesis

The effectiveness of exercise therapy was estimated for pre- and post-exercise (training intervention) change in HR, SV, and/or QOL for individuals with POTS in the included studies, as summarized in Figures 2, 3, where the effect direction of each outcome (clinically beneficial vs. non-beneficial) is summarized for each of the included reports. All included reports reported one or more HR-based outcome; of these, all reported positive (beneficial) direction of effect (*p* = 0.0078, two-tailed sign test). Three of the reports reported SV as an outcome; of these, all showed a positive direction of effect, (*p* = 0.250, two-tailed sign test). Finally, 4 reports reported a QOL based outcome measure; of these, all 4 reported a positive effect direction (*p* = 0.0625, two-tailed sign test).

### Reporting biases and certainty of evidence

As described in our Methods, our registered protocol (12) included the application of risk of bias evaluations to provide GRADE ratings in service of supporting practice recommendations (19). However, based on our risk of bias assessment, we elected not to perform formal GRADE assignments, given the overall level ofbias across studies. This factor critically limits the certainty of evidence, and as a result, clinical practice recommendations or specific exercise protocols could not be developed based on the results of this review.

## Discussion

The aim of this systematic review was to summarize and evaluate the literature related to therapeutic exercise as a treatment modality for patients with POTS. In addition to assessing overall clinical benefit, we also aimed to understand the extent to which prior studies considered participant selection, related comorbid conditions, and treatment factors (e.g., drop-out, adherence, tolerability, exercise protocol components). Our findings support the widely held assumption that therapeutic exercise can improve HR parameters, cardiac function, and QOL in individuals with POTS (Figures 2, 3). However, the majority of the reviewed studies were relatively small in size (all but two of had *n* < 50 subjects), often lacked *a priori* power analyses to determine sample size, and typically drew upon highly selected populations. As a result, nearly all studies (except one) suffered from moderate to high risk of bias and study design limitations (Figure 2). In particular, those with comorbid conditions that impact exercise tolerability remain severely understudied. Such factors also contribute to a key limitation of this review, including rendering the data inappropriate for meta-analysis. Finally, the heterogeneity of the included studies also limits the value of the collective data in terms of determining the ideal exercise prescription for clinical use.

Thus, a key conclusion of this review is to guide future research, which can provide further resolution as to the differential impact of exercise for patients with POTS and various comorbidities, which might alter exercise responses. Here we discuss several resulting themes related to the overall findings, quality of data, critical gaps and opportunities for future research.

### Study design and analysis approaches

Variable enrollment criteria, reporting of drop-out rates, adherence to study intervention, and screen failures limits interpretability and generalizability of the body of evidence in favor of therapeutic exercise for POTS. While two of the studies evaluated were RCTs, (one of which utilized a 30 minute tilt challenge rather than the consensus-based 10 min tilt (24)), the remaining studies were non-randomized case–control or pragmatic trials with four of the seven studies (20–23) appearing to be different analyses of the same dataset. Of these, drop-out rates were reported inconsistently and, where reported, varied quite widely (10–59%). Similarly, adherence was underreported (21, 25, 26), effectively limiting our understanding of the sustainability and tolerability of the interventions studied. Critically, the irregular reporting of screening failures and reasons for dropout further limits our understanding of barriers to exercise within individuals with POTS, and the viability and efficacy of various protocol attributes cannot be fully assessed. As an exception, one of the only studies to implement a semi-supervised exercise protocol (27) had one of the lowest reported dropout rates (16%), suggesting the context and/or setting the therapeutic exercise prescription (e.g., supervised vs. self-led) may also differentially influence adherence.

Several of the studies in this review used per-protocol (PP) and last observation carried forward (LOCF) analyses, rather than intention-to-treat (ITT), which contributed to the moderate to high risk of bias across studies. For context, PP analyses compare only those who complete their assigned treatment arm, and do not necessarily account for dropouts caused by the treatment itself (i.e., exercise intolerance). In a LOCF analysis, missing follow-up visits are replaced by that participant’s previously observed value (i.e., the last observation is carried forward); thus, the combination of the observed and imputed data is included in the final analysis as though there were no missing data. These approaches are commonly used in exercise trials since they allow for inclusion of only those that complete/receive the intended treatment. In contrast, ITT evaluates treatment outcomes of all participants originally allocated after randomization, regardless of whether they completed the study, or not. This has the advantage of capturing the treatment effect in a way that integrates dropout, which occurs in the real world. However, even ITT cannot account for adherence/tolerability factors that are related to medications which are taken in addition to (or instead of) exercise, and/or the implementation of lifestyle components (such as salt/hydration), which introduce still more variability in terms of estimating adherence and benefit to exercise as an intervention in POTS.

### Study population and outcome measures

As a syndrome likely stemming from multiple underlying and/or overlapping pathologies, POTS is widely accepted to be a heterogeneous disorder with multiple concurrent comorbidities, many of which impact symptom burden and may comingle with disease expression (29). However, the vast majority of the studies included in this review did not include even a crude comparison of idiopathic (POTS without comorbidities) vs. POTS with comorbidities. Clinically, POTS is diagnostically defined by a rise in HR on orthostatic challenge (4, 5, 30). However, this seemingly hallmark feature may be inconsistently present at the diagnostic and/or subsequent visits, and it is unknown as to whether improvement in HR alone directly results in improvements in overall function. Indeed, many patients with clinically impactful chronic orthostatic intolerance will not demonstrate postural tachycardia consistently (2), and diurnal variation in heart rate response to orthostatic challenge has been demonstrated (31). Vernino et al.’s recent clinical trial of intravenous immunoglobulin therapy reflects the variability of using HR criteria across timepoints in their cohort of participants with clinically confirmed POTS (32). Furthermore, most patients with POTS have a wide variety of non-HR related symptoms, which may comprise significant components of their symptom burden and impact function/QOL (7, 8, 33). These factors underlie a key concern about clinical trial outcomes relative to exercise as a treatment in POTS, and an over emphasis of HR-specific measures, with few studies attempting to capture functional outcomes. While all the reports included in this review demonstrated an improvement in HR and/or cardiac function, only a few evaluated multi-systemic symptoms and/or QOL stemming from signs/symptoms beyond cardiac specific manifestations, and none included a disease-relevant functional impact or disability measure.

Notably, HR changes were indeed the most robust physiological outcome reported across studies. Whereas, SV improvements were relatively modest (Figure 3). Given that CO is determined by a combination of HR and SV, the former being more variable, it is not surprising that any positive observed changes in SV were smaller than those in HR. While adaptive changes in resting heart rate, stroke volume, plasma volume, and left ventricular size occur with exercise training in healthy individuals, and limited data suggests that exercise training can modify these in patients with POTS (21), it is less clear whether these metrics can be truly normalized with exercise training for individuals with POTS. It is also possible that the exercise interventions themselves were not intense enough, nor long enough in duration, to affect a statistically significant change in SV (34).

Another key factor related to subject selection that was observed in this review was a general lack of consideration of comorbidities that could potentially impact exercise prescription and response. It has been reported that over 80% of individuals with POTS exhibit at least one comorbid diagnosis that could influence the approach to exercise – including but not limited to migraine headaches (40%), autoimmune diseases (9–16%), and hypermobility (25–61%) (35, 36). While there is some evidence that people with joint hypermobility may experience improved function with exercise, pain is a commonly reported limiting factor in their ability to participate in various types of exercise (37). In our review, the majority of studies *excluded* subjects with these key comorbidities, or did not consider comorbidities at all in their reported recruitment approach (20–23, 25), which further contributes to risk of bias. Similarly, studies that rely on populations that are dissimilar to the demographics and co-morbidties associated with POTS, or that apply variable diagnostic criteria, risk misleading conclusions as a result. For example, Winker et al. reported on an all-male cohort of soldier recruits actively serving in the Austrian army with idiopathic orthostatic intolerance, while 85–95% of POTS patients are female (24); and Gibbons, et al., reported that none of their study subjects were diagnosed with or suspected of having MCAS, while other investigators have reported over 60% of patients with POTS having one or more biomarker and/or symptoms suggestive of MCAS (38). Critically, these limitations across studies call into question the real-world generalizability of the reviewed literature. As notable exceptions, Gibbons et al. (25) and Wheatley-Guy et al. (27) were the only reports to include individuals with a POTS diagnosis while also allowing for hypermobility; similarly, Wheatley-Guy et al. (27) was the only study to specify inclusion of participants with POTS alongside a spectrum of relevant comorbidities including migraines/headache, MCAS, asthma, fatigue, pain, IBS, anemia and Sjögren’s disease, reflective of a population more commonly encountered in the clinical setting. Gibbons et al. also reported that participants with POTS and hypermobile EDS showed both improvement and minimal adverse effects with the exercise protocol, suggesting that exercise therapy may be potentially safe and tolerable in this setting. Further, there is some indication that supervised physical therapy might help address the limitations related to pain in this population (39). Future studies might further explore the potential role of individualized and/or adaptive exercise prescriptions (40) to mitigate the musculoskeletal pain and risk of injury associated with comorbidities that may contribute to higher dropout rates in exercise programs for this population. Even without formally adapted exercise programs, future studies could include more rudimentary subgroup analyses distinguishing between POTS populations with and without comorbid conditions.

Finally, it is estimated that a fair proportion of patients with POTS also meet the current diagnostic criteria for ME/CFS (41–43), which is also characterized by multisystemic symptomatology including autonomic dysfunction, and is exemplified by moderate to severe PEM. PEM is, by definition, precipitated by exertion (e.g., mental, emotional, or physical), and can result in a worsening of a constellation of symptoms, leading to extreme fatigue, cognitive impairment and flu-like symptoms within hours or days after exertion lasting days to weeks. Notably, none of the studies characterized (or considered) the participants prior experiences with exercise, which may influence an individual’s decision to participate in an exercise study. Since exercise is commonly recommended as a treatment for POTS, it is likely that most have tried some type of exercise prior to encountering an opportunity to enroll in a study. Those who have seen no benefit from exercise, or in some cases may feel worse after exercise (such as those with significant PEM), may be inherently less likely to volunteer for exercise studies, whereas patients who have seen some benefit from exercise are more likely to volunteer for exercise studies. This may itself, lead to further [self-]selection bias.

### Components of a therapeutic exercise prescription

A key, unexplored limitation encountered during this review was the wide variability of implemented exercise interventions, and while studies shared some common themes, there were a number of variations across the studies reviewed. The most common exercise intervention duration was 3 months, but Gibbons et al. (25) used a 6-month intervention period. Some programs were self-administered, while others were supervised. Protocols generally sought to gradually progress orthostatic load (progress from recumbent to more upright body position over time) as well as intensity, but the manner in which that was achieved varied. For example, the reports including data from Fu et al. (21), reference a protocol in which periodization was also progressed, starting with only 1–2 weekly sessions, ending with 4 weekly sessions, and including recovery sessions in between. Uniquely, this group also calculated training impulse or training load (TRIMP), although it was not a reported outcome measure, and not correlated to any symptomatology. This is a contrast to Gibbons et al. (25) where participants exercised 6 days/week, regardless of how other variables progressed. Of note, in this study, session duration appeared to progress without specific intensity guidance, where other studies, like Fu et al. (21) established HR-based intensities after a treadmill-based exercise capacity assessment. Some programs included strength training while others focused purely on cardiovascular endurance. It is not always made clear how rigidly these progressions were made. Certainly, any precipitated episodes of exercise intolerance or resulting drop-out is unclear, when some of these variables might be tied to meaningful clinical outcomes, whether positive or negative.

For the purposes of this review, any study deploying a structured aerobic exercise intervention of any kind was included. However, it is unknown to what extent varying protocols are equivalent. Like any prescribed treatment, dose is critical – necessitating reference to frequency, intensity, time and type (i.e., the “FITT” parameters) in order to comprise a complete exercise prescription (44). Of the studies that provided details regarding the FITT parameters of their intervention, the studied exercise protocols tended to vary widely across studies in how the parameters were applied to create a graded exercise program. It is noteworthy that the beneficial effects of exercise on cardiopulmonary parameters, such as decreased HR, increased SV, and improved $\dot{V}$ O_2_max, are essentially the same between POTS and healthy controls (21). These changes are likely the results of generalizable impacts of cardiopulmonary training. Fu et al. (21) tested the hypothesis that reduced blood volume contributes to POTS by comparing pre- and post-exercise intervention, which is known to increase blood volume. However, beyond this, few studies evaluate POTS-specific changes that occur in response to exercise. While a few studies in our review did evaluate changes in renin-angiotensin system and arterial baroreflex pathways, other mechanisms are known to be altered in POTS that could be influenced by exercise including changes in neuroendocrine, metabolic, hormonal and immune pathways, each meriting additional investigation as to potential POTS-specific mechanisms of exercise benefit. Several studies using invasive cardiopulmonary exercise testing have shown that participants with POTS and ME/CFS have reduced venous return during exercise when compared with healthy controls (45–47). In summary, existing studies suggest that aerobic exercise has beneficial effects on individuals with POTS, primarily by expanding blood volume and increasing venous return to the heart. However, it remains uncertain whether there are additional exercise effects specific to the underlying pathophysiology of POTS beyond the symptom suppressing effect of blood volume expansion and increased venous return, such as regrowth of small fiber nerves, or reduction in inflammatory cytokines, mast cell biomarkers or autoantibodies (e.g., neuroplasticity centrally and/or peripherally). This may require a better understanding of the etiological pathology of POTS in order to better understand. In the meantime, in the absence of clearer evidence-based guidance, in the setting of acute exercise intolerance (colloquially known as a “flare”), common practice points to using a combination of relative reduction in activity/training load, medication, and sufficient or increased sodium/water prescription.

### Opportunities for future research

In addition to the limitations noted above, and despite it being a current mainstay of clinical care, there remains a lack of data for the real-world use of exercise for the treatment of POTS. Many lingering questions remain: How long does one need to exercise to get benefit? Should the “dose” of exercise change over time? What happens if one stops exercise, will their POTS worsen? How does overtraining impact POTS symptoms and function? What are the potentially harmful effects of overtraining or undertraining in POTS? Data on long-term outcomes are extremely limited. To date, there is a paucity of information on outcomes beyond 3–6 months after an initial exercise intervention with respect to tolerability, symptom suppression, QOL, and functional status, in addition to sufficient quality of evidence to guide clinical best practices in terms of protocol selection. Anecdotally, based on the collective experience of the authors, many patients report quickly reverting to more symptomatic POTS if they stop engaging in regular aerobic exercise even for a few days. Additionally, some patients might even report a worsening of POTS symptoms that no longer respond to their previously effective exercise intervention during “POTS flares” or even gradually over time. Flares can be triggered by infections, concussions, pregnancy, surgeries, accidents/bodily injuries, vaccinations, periods of extreme emotional stress, and other events, including over-exercise (intensity, frequency, progression of volume all being potential culprit variables) (48, 49). All of these factors merit our continued attention and ongoing study.

## Conclusion

Despite the overall high risk of bias present in the available reports, and the relatively low volume of literature to review, available literature supports a role for therapeutic exercise for the treatment of POTS. Future studies should build upon the above identified weaknesses in study design and structure to include: (1) adequately powered studies (ideally multi-center allowing for larger recruitment numbers), (2) prospective pre-enrollment characterization of co-morbidities that may influence exercise outcomes and reporting of treatment response relative to pertinent co-morbidity related metrics, (3) longer follow-up intervals, (4) consideration of semi-supervised exercise interventions, (5) detailed intervention protocols that allow for standardization and dissemination of exercise programming variables, (6) better characterization of functional, activities of daily living, and non-orthostatic symptom outcomes, as well as physiological and fluid biomarkers known to be abnormal in POTS, and (7) transparent reporting of patient-reported reasons for screen failure, dropout and non-adherence.

## Footnotes

^1^ www.graphpad.com/quickcalcs/binomial1

^2^https://jbi.global/critical-appraisal-tools, accessed 17 June, 2024.
