# Supplementary material for: Impact of exercise to treat postural orthostatic tachycardia syndrome: a systematic review
Source: Front Neurol. 2025 Apr 24;16:1567708. doi: 10.3389/fneur.2025.1567708 (PMC12071195; doi:10.3389/fneur.2025.1567708)
Supplement: Supplementary file 1 [file Table_1.docx]

**Supplement 1**

**Strategies for POTS-Exercise systematic review**

Databases:

Medline (Ovid) 1946-2023, Embase (Elsevier) 1974 - 2023, CINAHL Complete (Ebscohost) 1937-2023, Cochrane CENTRAL (Wiley) 1898-2023, APA PsycINFO (Ebscohost) 1872-2023, Psychology and Behavioral Sciences Collection (Ebscohost) dates vary by title, SportDiscus (Ebscohost) 1800 – 2023, Scopus (Elsevier) 1970-2023 and Web of Science Core Collection (Clarivate) 1900-2023.

- No date nor methodology filters will be applied to databases, although conference abstracts will be excluded from Embase, Scopus and Web of Science.
- All searches were ran and results exported on May 2, 2023
- Legend for field codes and proximity operators defined at end of strategy for each database.

**Ovid MEDLINE(R)** May 2, 2023

1 Postural Orthostatic Tachycardia Syndrome/ or ("orthostatic tachycardia*" or "postural tachycardia*" or "postural tachycardia syndrome" or "postural orthostatic tachycardia syndrome" or ((orthostatic or postural) adj3 (tachycardia* or POTS))).ti,ab,kf,kw. [POTS subject/keywords] 1596

2 Orthostatic Intolerance/ or ("orthostatic intolerance" or "orthostatic dysregulation").ti,ab,kf,kw. [Orthostatic intolerance subject/keyword ] 1844

3 exercise therapy/ or blood flow restriction therapy/ or endurance training/ or motion therapy, continuous passive/ or muscle stretching exercises/ or plyometric exercise/ or resistance training/ [exercise therapy subject terms] 62727

4 ((exercise* adj2 (intervention* or method* or procedure* or technique* or technic* or therap* or treatment*)) or ((bfr or "blood flow restriction*") adj2 (exercise* or therap* or training)) or (endurance adj1 (exercise* or training)) or "closed kinetic chain exercise" or "open kinetic chain exercise*" or "dynamic exercise" or "exercise recovery" or "recovery exercise" or "isokinetic exercise*" or "isometric exercise*" or "isometric endurance" or "isometric training" or "static exercise*" or ((CPM or "continuous passive motion" or "continuous passive movement") adj2 (therapy or therapies)) or "muscle training" or "muscle strengthening" or "myofunctional therapy" or ((active or ballistic or dynamic or isometric or PNF or passive or relaxed or "static active" or static-active or "static passive" or static-passive or static) adj2 (stretching or stretchings)) or "Neuromuscular Facilitation*" or (("cycle exercise*" or drill or drills or exercise*) adj2 (stretch-shortening or "stretch shortening")) or plyometric or plyometrics or "pelvic floor muscle training" or "Kegel exercise*" or "pelvic floor exercise*" or "pelvic floor muscle exercise*" or "pelvic floor training" or "pelvic muscle exercise*" or "pelvic muscle training" or ((resistance or strength) adj2 training) or ((weight-bearing or " weight bearing" or weight-lifting or "weight lifting") adj2 ("exercise program*" or " strengthening program*"))).ti,ab,kf,kw. [exercise therapy keywords] 67227

5 Aquatic Therapy/ or ((aquatic or "Ai Chi" or pool or water or "water tai chi") adj2 therapy).ti,ab,kf,kw. [aquatic therapy subject/keyword terms] 828

6 (kinesiotherap* or kinesitherap* or "corrective exercise*" or "exercise movement technique*" or "exercise therapy" or "exercise treatment*" or "therapeutic exercise").ti,ab,kf,kw. [exercise therapy keywords 2] 9171

7 or/3-6 [Exercise/Aquatic therapy set] 107934

8 exercise movement techniques/ or breathing exercises/ or qigong/ or dance therapy/ or tai ji/ or yoga/ [Exercise movement techniques - EMT subject terms] 10203

9 ("exercise movement" or "movement therapy" or "motion therapy" or pilates or pilates-based or "breath* exercise*" or "breath* therapy" or "breath* work" or "holotropic breathing" or "Buteyko breathing" or "Buteyko method" or "Buteyko training" or Pranayama or "yoga breathing" or "yogic breathing" or "respiratory muscle training" or Qigong or "ch'i kung" or "qi gong" or (dance adj1 therap*) or "Tai ji" or "t'ai chi" or "tai chi" or "tai ji" or tai-ji or taiji or taijiquan or Yoga).ti,ab,kf,kw. [Exercise Movement Techniques -subject/keywords] 13748

10 or/8-9 [Exercise Movement set] 17382

11 exercise/ or cool-down exercise/ or exergaming/ or gymnastics/ or muscle stretching exercises/ or physical conditioning, animal/ or physical conditioning, human/ or circuit-based exercise/ or endurance training/ or high-intensity interval training/ or plyometric exercise/ or resistance training/ or preoperative exercise/ or running/ or jogging/ or marathon running/ or swimming/ or walking/ or nordic walking/ or stair climbing/ or warm-up exercise/ [Exercise subject terms] 244214

12 (exercise or exercises or aerobic* or "biometric exercise" or "exercise capacity" or "exercise performance" or "exercise training" or "exertion" or "fitness training" or "fitness workout" or "physical conditioning, human" or "physical effort" or "physical exercise" or "physical exertion" or "physical work-out" or "physical workout" or "physical activit*" or Exergam* or (("active video" or active-video) adj2 gaming*) or ("virtual reality" adj1 exercis*) or gymnastics or calisthenics or "physical conditioning" or "physical training" or (("circuit based" or circuit-based or circuit-type) adj1 exercise*) or "circuit training" or "endurance training" or ((high-intensity or "high intensity") adj2 ("interval training*" or "intermittent training*" or "intermittent exercise*")) or "sprint interval training*" or plyometric* or plyometry or ((stretch-shortening or "stretch shortening") adj1 (drill* or exercise* or "cycle exercise*")) or ((pre-operative or preoperative) adj1 (conditioning or exercise* or rehabilitation*)) or prehabilitation or running or jogging or swimming or walking or ambulation or "stair climbing" or "stair navigation" or ((warm-up or warmup or "warm up" or cool-down or cooling-down or "cool* down" or cooldown or "cooling down" or coolingdown or warmdown or warm-down or warming-down or "warming down") adj1 exercise*)).ti,ab,kf,kw. [exercise keywords] 712524

13 or/11-12 [Exercise set] 755952

14 Physical Therapy Modalities/ or (physiotherap* or "physical therap*").ti,ab,kf,kw. [Physical therapy subject/keyword terms] 84474

15 Yoga/ or yoga.ti,ab,kf,kw. [Yoga subject/keywords] 7048

16 Reflex, Stretch/ or ((reflex or tendon) adj1 stretch*).ti,ab,kf,kw. [Stretch reflex subject/keywords] 4529

17 "Range of Motion, Articular"/ or ("range of motion" or "joint flexibility").ti,ab,kf,kw. [Range of Motion - ROM subject/keywords] 83344

18 Telerehabilitation/ or (Telerehabilitation* or "tele rehabilitation*" or tele-rehabilitation* or ((remote or virtual) adj1 rehabilitation*)).ti,ab,kf,kw. [Telerehabilitation subject/keywords] 2595

19 Physical Exertion/ or (physical adj1 (exertion or effort)).ti,ab,kf,kw. [Physical exertion subject/keywords] 60094

20 Physical Endurance/ or (physical adj2 (endurance or stamina)).ti,ab,kf,kw. [Physical endurance subject/keywords] 21111

21 ("25487551" or "33482385" or "20579544" or "26690066").ui. [4 exemplar studies] 4

22 (or/1-2) and 7 [POTS + Exercise/Aquatic therapy set] 81

23 (or/1-2) and 10 [POTS + Exercise movement set] 7

24 (or/1-2) and 13 [POTS + Exercise set] 405

25 (or/1-2) and 14 [POTS + Physical Therapy] 23

26 (or/1-2) and 15 [POTS + Yoga set] 4

27 (or/1-2) and 16 [POTS + Stretch reflex set] 1

28 (or/1-2) and 17 [POTS + ROM set] 5

29 (or/1-2) and 18 [POTS + Telerehabilitation] 1

30 (or/1-2) and (or/19-20) [POTS + Physical exertion/Physical endurance] 40

31 or/22-30 [Final set] **442**

Medline (Ovid) legend:

Field codes: / = Medical Subject Heading (MeSH); ti = title; ab = abstract; kf = keyword heading word; kw = keyword heading (author keywords)

Proximity operator: adj#

Truncation: *

**Embase (Elsevier, embase.com)**  May 2, 2023

#14 #12 NOT ('conference abstract'/it OR 'conference review'/it) **701**

#13 #3 AND #9 934

#12 #10 OR #11 934

#11 #2 AND #9 NOT #10 350

#10 #1 AND #9 584

#9 #4 OR #5 OR #6 OR #7 OR #8 1162964

#8 'telerehabilitation'/de OR telerehabilitation*:ti,ab,kw OR 'tele rehabilitation*':ti,ab,kw OR (((remote OR virtual) NEAR/1 rehabilitation*):ti,ab,kw) 3390

#7 (((reflex OR tendon) NEAR/1 stretch*):ti,ab,kw) OR 'range of motion':ti,ab,kw OR 'joint flexibility':ti,ab,kw OR ((physical NEAR/1 (exertion OR effort)):ti,ab,kw) OR ((physical NEAR/2 (endurance OR stamina)):ti,ab,kw) 62452

#6 'exercise'/exp OR exercise:ti,ab,kw OR exercises:ti,ab,kw OR aerobic*:ti,ab,kw OR 'biometric exercise':ti,ab,kw OR 'exercise capacity':ti,ab,kw OR 'exercise performance':ti,ab,kw OR 'exercise training':ti,ab,kw OR 'exertion':ti,ab,kw OR 'fitness training':ti,ab,kw OR 'fitness workout':ti,ab,kw OR 'physical conditioning, human':ti,ab,kw OR 'physical effort':ti,ab,kw OR 'physical exercise':ti,ab,kw OR 'physical exertion':ti,ab,kw OR 'physical work-out':ti,ab,kw OR 'physical workout':ti,ab,kw OR 'physical activit*':ti,ab,kw OR exergam*:ti,ab,kw OR ((('active video' OR 'active video') NEAR/2 gaming*):ti,ab,kw) OR (('virtual reality' NEAR/1 exercis*):ti,ab,kw) OR gymnastics:ti,ab,kw OR calisthenics:ti,ab,kw OR 'physical conditioning':ti,ab,kw OR 'physical training':ti,ab,kw OR ((('circuit based' OR 'circuit based' OR 'circuit type') NEAR/1 exercise*):ti,ab,kw) OR 'circuit training':ti,ab,kw OR 'endurance training':ti,ab,kw OR ((('high intensity' OR 'high intensity') NEAR/2 ('interval training*' OR 'intermittent training*' OR 'intermittent exercise*')):ti,ab,kw) OR 'sprint interval training*':ti,ab,kw OR plyometric*:ti,ab,kw OR plyometry:ti,ab,kw OR ((('stretch shortening' OR 'stretch shortening') NEAR/1 (drill* OR exercise* OR 'cycle exercise*')):ti,ab,kw) OR ((('pre operative' OR preoperative) NEAR/1 (conditioning OR exercise* OR rehabilitation*)):ti,ab,kw) OR prehabilitation:ti,ab,kw OR running:ti,ab,kw OR jogging:ti,ab,kw OR swimming:ti,ab,kw OR walking:ti,ab,kw OR ambulation:ti,ab,kw OR 'stair climbing':ti,ab,kw OR 'stair navigation':ti,ab,kw OR ((('warm up' OR warmup OR 'warm up' OR 'cool down' OR 'cooling down' OR 'cool* down' OR cooldown OR 'cooling down' OR coolingdown OR warmdown OR 'warm down' OR 'warming down' OR 'warming down') NEAR/1 exercise*):ti,ab,kw) 1044806

#5 'exercise'/exp OR exercise:ti,ab,kw OR exercises:ti,ab,kw OR aerobic*:ti,ab,kw OR 'biometric exercise':ti,ab,kw OR 'exercise capacity':ti,ab,kw OR 'exercise performance':ti,ab,kw OR 'exercise training':ti,ab,kw OR 'exertion':ti,ab,kw OR 'fitness training':ti,ab,kw OR 'fitness workout':ti,ab,kw OR 'physical conditioning, human':ti,ab,kw OR 'physical effort':ti,ab,kw OR 'physical exercise':ti,ab,kw OR 'physical exertion':ti,ab,kw OR 'physical work-out':ti,ab,kw OR 'physical workout':ti,ab,kw OR 'physical activit*':ti,ab,kw OR exergam*:ti,ab,kw OR ((('active video' OR 'active video') NEAR/2 gaming*):ti,ab,kw) OR (('virtual reality' NEAR/1 exercis*):ti,ab,kw) OR gymnastics:ti,ab,kw OR calisthenics:ti,ab,kw OR 'physical conditioning':ti,ab,kw OR 'physical training':ti,ab,kw OR ((('circuit based' OR 'circuit based' OR 'circuit type') NEAR/1 exercise*):ti,ab,kw) OR 'circuit training':ti,ab,kw OR 'endurance training':ti,ab,kw OR ((('high intensity' OR 'high intensity') NEAR/2 ('interval training*' OR 'intermittent training*' OR 'intermittent exercise*')):ti,ab,kw) OR 'sprint interval training*':ti,ab,kw OR plyometric*:ti,ab,kw OR plyometry:ti,ab,kw OR ((('stretch shortening' OR 'stretch shortening') NEAR/1 (drill* OR exercise* OR 'cycle exercise*')):ti,ab,kw) OR ((('pre operative' OR preoperative) NEAR/1 (conditioning OR exercise* OR rehabilitation*)):ti,ab,kw) OR prehabilitation:ti,ab,kw OR running:ti,ab,kw OR jogging:ti,ab,kw OR swimming:ti,ab,kw OR walking:ti,ab,kw OR ambulation:ti,ab,kw OR 'stair climbing':ti,ab,kw OR 'stair navigation':ti,ab,kw OR ((('warm up' OR warmup OR 'warm up' OR 'cool down' OR 'cooling down' OR 'cool* down' OR cooldown OR 'cooling down' OR coolingdown OR warmdown OR 'warm down' OR 'warming down' OR 'warming down') NEAR/1 exercise*):ti,ab,kw) 1044806

#4 'kinesiotherapy'/exp OR kinesiotherap*:ti,ab,kw OR kinesitherap*:ti,ab,kw OR 'corrective exercise*':ti,ab,kw OR 'exercise movement*':ti,ab,kw OR 'exercise therapy':ti,ab,kw OR 'exercise treatment*':ti,ab,kw OR 'therapeutic exercise':ti,ab,kw OR ((exercise* NEAR/2 (intervention* OR method* OR procedure* OR technique* OR technic* OR therap* OR treatment*)):ti,ab,kw) OR (((bfr OR 'blood flow restriction*') NEAR/2 (exercise* OR therap* OR training)):ti,ab,kw) OR ((endurance NEAR/1 (exercise* OR training)):ti,ab,kw) OR 'closed kinetic chain exercise':ti,ab,kw OR 'open kinetic chain exercise*':ti,ab,kw OR 'dynamic exercise':ti,ab,kw OR 'exercise recovery':ti,ab,kw OR 'recovery exercise':ti,ab,kw OR 'isokinetic exercise*':ti,ab,kw OR 'isometric exercise*':ti,ab,kw OR 'isometric endurance':ti,ab,kw OR 'isometric training':ti,ab,kw OR 'static exercise*':ti,ab,kw OR (((cpm OR 'continuous passive motion' OR 'continuous passive movement') NEAR/2 (therapy OR therapies)):ti,ab,kw) OR 'muscle training':ti,ab,kw OR 'muscle strengthening':ti,ab,kw OR 'myofunctional therapy':ti,ab,kw OR (((active OR ballistic OR dynamic OR isometric OR pnf OR passive OR relaxed OR 'static active' OR 'static active' OR 'static passive' OR 'static passive' OR static) NEAR/2 (stretching OR stretchings)):ti,ab,kw) OR 'neuromuscular facilitation*':ti,ab,kw OR ((('cycle exercise*' OR drill OR drills OR exercise*) NEAR/2 ('stretch shortening' OR 'stretch shortening')):ti,ab,kw) OR plyometric:ti,ab,kw OR plyometrics:ti,ab,kw OR 'kegel exercise*':ti,ab,kw OR 'pelvic floor exercise*':ti,ab,kw OR 'pelvic floor muscle exercise*':ti,ab,kw OR 'pelvic floor training':ti,ab,kw OR 'pelvic muscle exercise*':ti,ab,kw OR 'pelvic muscle training':ti,ab,kw OR (((resistance OR strength) NEAR/2 training):ti,ab,kw) OR ((('weight bearing' OR 'weight bearing' OR 'weight lifting' OR 'weight lifting') NEAR/2 ('exercise program*' OR 'strengthening program*')):ti,ab,kw) OR (((aquatic OR 'ai chi' OR pool OR water OR 'water tai chi') NEAR/2 therapy):ti,ab,kw) OR physiotherap*:ti,ab,kw OR 'physical thearp*':ti,ab,kw OR pilates:ti,ab,kw OR 'pilates based':ti,ab,kw OR 'breath* exercise*':ti,ab,kw OR 'breath* therapy':ti,ab,kw OR 'breath* work':ti,ab,kw OR 'holotropic breathing':ti,ab,kw OR 'buteyko breathing':ti,ab,kw OR 'buteyko method':ti,ab,kw OR 'buteyko training':ti,ab,kw OR pranayama:ti,ab,kw OR 'yoga breathing':ti,ab,kw OR 'yogic breathing':ti,ab,kw OR 'respiratory muscle training':ti,ab,kw OR qigong:ti,ab,kw OR 'ch?i kung':ti,ab,kw OR 'qi gong':ti,ab,kw OR ((dance NEAR/1 therap*):ti,ab,kw) OR 't?ai chi':ti,ab,kw OR 'tai chi':ti,ab,kw OR 'tai ji':ti,ab,kw OR taiji:ti,ab,kw OR taijiquan:ti,ab,kw OR yoga:ti,ab,kw 218813

#3 #1 OR #2 9426

#2 'orthostatic intolerance'/de OR 'orthostatic intolerance':ti,ab,kw OR 'orthostatic dysregulation':ti,ab,kw 3139

#1 'postural orthostatic tachycardia syndrome'/de OR 'orthostatic tachycardia*':ti,ab,kw OR 'postural tachycardia*':ti,ab,kw OR 'postural tachycardia syndrome':ti,ab,kw OR pots:ti,ab,kw OR 'postural orthostatic tachycardia syndrome':ti,ab,kw OR (((orthostatic OR postural) NEAR/3 tachycardia*):ti,ab,kw) 7304

Embase legend:

Field codes: ti = Title, ab = Abstract, kw = Keyword, de = Index term (Descriptor - combined drug and medical)

Proximity operator: NEAR/#

Truncation: *

**Cochrane Central Register of Controlled Trials - CENTRAL (Wiley.com)** May 2, 2023

ID Search Hits

#1 MeSH descriptor: [Postural Orthostatic Tachycardia Syndrome] this term only 94

#2 (("orthostatic tachycardia*" or "postural tachycardia*" or "postural tachycardia syndrome" or "postural orthostatic tachycardia syndrome" or ((orthostatic or postural) NEAR/3 (tachycardia* or POTS)))):ti,ab,kw 220

#3 #1 or #2 220

#4 MeSH descriptor: [Orthostatic Intolerance] this term only 69

#5 (("orthostatic intolerance" or "orthostatic dysregulation")):ti,ab,kw 257

#6 #4 or #5 257

#7 MeSH descriptor: [Exercise Therapy] explode all trees 19456

#8 (((exercise* NEAR/2 (intervention* or method* or procedure* or technique* or technic* or therap* or treatment*)) or ((bfr or "blood flow restriction*") NEAR/2 (exercise* or therap* or training)) or (endurance NEAR/1 (exercise* or training)) or "closed kinetic chain exercise" or "open kinetic chain exercise*" or "dynamic exercise" or "exercise recovery" or "recovery exercise" or "isokinetic exercise*" or "isometric exercise*" or "isometric endurance" or "isometric training" or "static exercise*" or ((CPM or "continuous passive motion" or "continuous passive movement") NEAR/2 (therapy or therapies)) or "muscle training" or "muscle strengthening" or "myofunctional therapy" or ((active or ballistic or dynamic or isometric or PNF or passive or relaxed or "static active" or static-active or "static passive" or static-passive or static) NEAR/2 (stretching or stretchings)) or "Neuromuscular Facilitation*" or (("cycle exercise*" or drill or drills or exercise*) NEAR/2 (stretch-shortening or "stretch shortening")) or plyometric or plyometrics or "pelvic floor muscle training" or "Kegel exercise*" or "pelvic floor exercise*" or "pelvic floor muscle exercise*" or "pelvic floor training" or "pelvic muscle exercise*" or "pelvic muscle training" or ((resistance or strength) NEAR/2 training) or ((weight-bearing or " weight bearing" or weight-lifting or "weight lifting") NEAR/2 ("exercise program*" or " strengthening program*")))):ti,ab,kw 57898

#9 #7 or #8 58157

#10 MeSH descriptor: [Aquatic Therapy] explode all trees 9

#11 (((aquatic or "Ai Chi" or pool or water or "water tai chi") NEAR/2 therapy)):ti,ab,kw 430

#12 #10 or #11 430

#13 ((kinesiotherap* or kinesitherap* or "corrective exercise*" or "exercise movement technique*" or "exercise therapy" or "exercise treatment*" or "therapeutic exercise")):ti,ab,kw 19333

#14 MeSH descriptor: [Exercise Movement Techniques] explode all trees 3183

#15 (("exercise movement" or "movement therapy" or "motion therapy" or pilates or pilates-based or "breath* exercise*" or "breath* therapy" or "breath* work" or "holotropic breathing" or "Buteyko breathing" or "Buteyko method" or "Buteyko training" or Pranayama or "yoga breathing" or "yogic breathing" or "respiratory muscle training" or Qigong or "ch'i kung" or "qi gong" or (dance NEAR/1 therap*) or "Tai ji" or "t'ai chi" or "tai chi" or "tai ji" or tai-ji or taiji or taijiquan or Yoga)):ti,ab,kw 10350

#16 #14 or #15 11103

#17 MeSH descriptor: [Exercise] explode all trees 38046

#18 ((exercise or exercises or aerobic* or "biometric exercise" or "exercise capacity" or "exercise performance" or "exercise training" or "exertion" or "fitness training" or "fitness workout" or "physical conditioning, human" or "physical effort" or "physical exercise" or "physical exertion" or "physical work-out" or "physical workout" or "physical activit*" or Exergam* or (("active video" or active-video) NEAR/2 gaming*) or ("virtual reality" NEAR/1 exercis*) or gymnastics or calisthenics or "physical conditioning" or "physical training" or (("circuit based" or circuit-based or circuit-type) NEAR/1 exercise*) or "circuit training" or "endurance training" or ((high-intensity or "high intensity") NEAR/2 ("interval training*" or "intermittent training*" or "intermittent exercise*")) or "sprint interval training*" or plyometric* or plyometry or ((stretch-shortening or "stretch shortening") NEAR/1 (drill* or exercise* or "cycle exercise*")) or ((pre-operative or preoperative) NEAR/1 (conditioning or exercise* or rehabilitation*)) or prehabilitation or running or jogging or swimming or walking or ambulation or "stair climbing" or "stair navigation" or ((warm-up or warmup or "warm up" or cool-down or cooling-down or "cool* down" or cooldown or "cooling down" or coolingdown or warmdown or warm-down or warming-down or "warming down") NEAR/1 exercise*))):ti,ab,kw 157368

#19 #17 or #18 158123

#20 MeSH descriptor: [Physical Therapy Modalities] explode all trees 35523

#21 ((physiotherap* or "physical therap*")):ti,ab,kw 21090

#22 #20 or #21 52435

#23 MeSH descriptor: [Yoga] this term only 1195

#24 (yoga):ti,ab,kw 4951

#25 #23 or #24 4951

#26 MeSH descriptor: [Telerehabilitation] this term only 272

#27 ((Telerehabilitation* or "tele rehabilitation*" or tele-rehabilitation* or ((remote or virtual) NEAR/1 rehabilitation*))):ti,ab,kw 1349

#28 #26 or #27 1349

#29 MeSH descriptor: [Physical Exertion] this term only 4238

#30 ((physical NEAR/1 (exertion or effort))):ti,ab,kw 4656

#31 #29 or #30 4656

#32 #3 AND (#6 OR #9 OR #12 OR #13 OR #16 OR #19 OR #22 OR #25 OR #28 OR #31) 87

#33 #32 in Trials 87

#34 #6 AND (#6 OR #9 OR #12 OR #13 OR #16 OR #19 OR #22 OR #25 OR #28 OR #31) 257

#35 #34 in Trials 257

#36 #32 OR #35 **281**

Cochrane Library legend:

Field codes: ti = Title, ab = Abstract, kw = Keyword, MeSH descriptor = Medical Subject Heading

Proximity operator: NEAR/#

Truncation:  *

**CINAHL Complete (EBSCOhost)** May 2, 2023

S6 S1 AND (S2 OR S3 OR S4 OR S5) **95**

S5 (MH "Exertion") OR (MH "Physical Endurance+") OR TI ( (physical N/1 (exertion or effort)) OR (physical N/2 (endurance or stamina)) ) OR AB ( (physical N/1 (exertion or effort)) OR (physical N/2 (endurance or stamina)) ) 21,082

S4 (MH "Physical Therapy+") OR (MH "Telerehabilitation") OR TI ( physiotherap* or "physical therap*" OR (Telerehabilitation* or "tele rehabilitation*" or tele-rehabilitation* or ((remote or virtual) N/1 rehabilitation*))(physical N/1 (exertion or effort)) OR (physical N/2 (endurance or stamina)) OR hydrotherap* OR ((aquatic or "Ai Chi" or pool or water or "water tai chi") N/2 therapy) ) OR AB ( physiotherap* or "physical therap*" OR (Telerehabilitation* or "tele rehabilitation*" or tele-rehabilitation* or ((remote or virtual) N/1 rehabilitation*))(physical N/1 (exertion or effort)) OR (physical N/2 (endurance or stamina)) OR hydrotherap* OR ((aquatic or "Ai Chi" or pool or water or "water tai chi") N/2 therapy) )

185,863

S3 (MH "Exercise+") OR TI ( (exercise or exercises or aerobic* or "biometric exercise" or "exercise capacity" or "exercise performance" or "exercise training" or "exertion" or "fitness training" or "fitness workout" or "physical conditioning, human" or "physical effort" or "physical exercise" or "physical exertion" or "physical work-out" or "physical workout" or "physical activit*" or Exergam* or (("active video" or active-video) N/2 gaming*) or ("virtual reality" N/1 exercis*) or gymnastics or calisthenics or "physical conditioning" or "physical training" or (("circuit based" or circuit-based or circuit-type) N/1 exercise*) or "circuit training" or "endurance training" or ((high-intensity or "high intensity") N/2 ("interval training*" or "intermittent training*" or "intermittent exercise*")) or "sprint interval training*" or plyometric* or plyometry or ((stretch-shortening or "stretch shortening") N/1 (drill* or exercise* or "cycle exercise*")) or ((pre-operative or preoperative) N/1 (conditioning or exercise* or rehabilitation*)) or prehabilitation or running or jogging or swimming or walking or ambulation or "stair climbing" or "stair navigation" or ((warm-up or warmup or "warm up" or cool-down or cooling-down or "cool* down" or cooldown or "cooling down" or coolingdown or warmdown or warm-down or warming-down or "warming down") N/1 exercise*)) ) OR AB ( (exercise or exercises or aerobic* or "biometric exercise" or "exercise capacity" or "exercise performance" or "exercise training" or "exertion" or "fitness training" or "fitness workout" or "physical conditioning, human" or "physical effort" or "physical exercise" or "physical exertion" or "physical work-out" or "physical workout" or "physical activit*" or Exergam* or (("active video" or active-video) N/2 gaming*) or ("virtual reality" N/1 exercis*) or gymnastics or calisthenics or "physical conditioning" or "physical training" or (("circuit based" or circuit-based or circuit-type) N/1 exercise*) or "circuit training" or "endurance training" or ((high-intensity or "high intensity") N/2 ("interval training*" or "intermittent training*" or "intermittent exercise*")) or "sprint interval training*" or plyometric* or plyometry or ((stretch-shortening or "stretch shortening") N/1 (drill* or exercise* or "cycle exercise*")) or ((pre-operative or preoperative) N/1 (conditioning or exercise* or rehabilitation*)) or prehabilitation or running or jogging or swimming or walking or ambulation or "stair climbing" or "stair navigation" or ((warm-up or warmup or "warm up" or cool-down or cooling-down or "cool* down" or cooldown or "cooling down" or coolingdown or warmdown or warm-down or warming-down or "warming down") N/1 exercise*)) ) 301,145

S2 (MH "Therapeutic Exercise+") OR TI ( kinesiotherap* or kinesitherap* or "corrective exercise*" or "exercise movement technique*" or "exercise therapy" or "exercise treatment*" or "therapeutic exercise" OR ((exercise* N/2 (intervention* or method* or procedure* or technique* or technic* or therap* or treatment*)) or ((bfr or "blood flow restriction*") N/2 (exercise* or therap* or training)) or (endurance N/1 (exercise* or training)) or "closed kinetic chain exercise" or "open kinetic chain exercise*" or "dynamic exercise" or "exercise recovery" or "recovery exercise" or "isokinetic exercise*" or "isometric exercise*" or "isometric endurance" or "isometric training" or "static exercise*" or ((CPM or "continuous passive motion" or "continuous passive movement") N/2 (therapy or therapies)) or "muscle training" or "muscle strengthening" or "myofunctional therapy" or ((active or ballistic or dynamic or isometric or PNF or passive or relaxed or "static active" or static-active or "static passive" or static-passive or static) N/2 (stretching or stretchings)) or "Neuromuscular Facilitation*" or (("cycle exercise*" or drill or drills or exercise*) N/2 (stretch-shortening or "stretch shortening")) or plyometric or plyometrics or "pelvic floor muscle training" or "Kegel exercise*" or "pelvic floor exercise*" or "pelvic floor muscle exercise*" or "pelvic floor training" or "pelvic muscle exercise*" or "pelvic muscle training" or ((resistance or strength) N/2 training) or ((weight-bearing or " weight bearing" or weight-lifting or "weight lifting") N/2 ("exercise program*" or " strengthening program*"))) ) OR AB ( kinesiotherap* or kinesitherap* or "corrective exercise*" or "exercise movement technique*" or "exercise therapy" or "exercise treatment*" or "therapeutic exercise" OR ((exercise* N/2 (intervention* or method* or procedure* or technique* or technic* or therap* or treatment*)) or ((bfr or "blood flow restriction*") N/2 (exercise* or therap* or training)) or (endurance N/1 (exercise* or training)) or "closed kinetic chain exercise" or "open kinetic chain exercise*" or "dynamic exercise" or "exercise recovery" or "recovery exercise" or "isokinetic exercise*" or "isometric exercise*" or "isometric endurance" or "isometric training" or "static exercise*" or ((CPM or "continuous passive motion" or "continuous passive movement") N/2 (therapy or therapies)) or "muscle training" or "muscle strengthening" or "myofunctional therapy" or ((active or ballistic or dynamic or isometric or PNF or passive or relaxed or "static active" or static-active or "static passive" or static-passive or static) N/2 (stretching or stretchings)) or "Neuromuscular Facilitation*" or (("cycle exercise*" or drill or drills or exercise*) N/2 (stretch-shortening or "stretch shortening")) or plyometric or plyometrics or "pelvic floor muscle training" or "Kegel exercise*" or "pelvic floor exercise*" or "pelvic floor muscle exercise*" or "pelvic floor training" or "pelvic muscle exercise*" or "pelvic muscle training" or ((resistance or strength) N/2 training) or ((weight-bearing or " weight bearing" or weight-lifting or "weight lifting") N/2 ("exercise program*" or " strengthening program*"))) OR TI ( ("exercise movement" or "movement therapy" or "motion therapy" or pilates or pilates-based or "breath* exercise*" or "breath* therapy" or "breath* work" or "holotropic breathing" or "Buteyko breathing" or "Buteyko method" or "Buteyko training" or Pranayama or "yoga breathing" or "yogic breathing" or "respiratory muscle training" or Qigong or "ch'i kung" or "qi gong" or (dance N/1 therap*) or "Tai ji" or "t'ai chi" or "tai chi" or "tai ji" or tai-ji or taiji or taijiquan or Yoga) ) OR AB ( ("exercise movement" or "movement therapy" or "motion therapy" or pilates or pilates-based or "breath* exercise*" or "breath* therapy" or "breath* work" or "holotropic breathing" or "Buteyko breathing" or "Buteyko method" or "Buteyko training" or Pranayama or "yoga breathing" or "yogic breathing" or "respiratory muscle training" or Qigong or "ch'i kung" or "qi gong" or (dance N/1 therap*) or "Tai ji" or "t'ai chi" or "tai chi" or "tai ji" or tai-ji or taiji or taijiquan or Yoga) )

78,145

S1 (MH "Postural Orthostatic Tachycardia Syndrome") OR (MH "Orthostatic Intolerance") OR TI ( ("orthostatic tachycardia*" or "postural tachycardia*" or "postural tachycardia syndrome" or "postural orthostatic tachycardia syndrome" or ((orthostatic or postural) N/3 (tachycardia* or POTS))) ) OR AB ( ("orthostatic tachycardia*" or "postural tachycardia*" or "postural tachycardia syndrome" or "postural orthostatic tachycardia syndrome" or ((orthostatic or postural) N/3 (tachycardia* or POTS))) ) 666

CINAHL legend:

Field codes: TI = title, AB= abstract, MH = CINAHL Exact Subject Headings

Proximity operator: N#

Truncation: *

**APA PsycInfo (EBSCOhost**) May 2, 2023

S6 S1 AND (S2 OR S3 OR S4 or S5) **17**

S5 ( (DE "Physical Endurance" OR DE "Physical Strength") OR TI ( (physical N/1 (exertion or effort)) OR (physical N/2 (endurance or stamina)) ) OR AB ( (physical N/1 (exertion or effort)) OR (physical N/2 (endurance or stamina)) ) ) OR KW ( (physical N/1 (exertion or effort)) OR (physical N/2 (endurance or stamina)) ) 3,303

S4 ( (DE "Physical Therapy") OR (DE "Telerehabilitation") OR TI ( physiotherap* or "physical therap*" OR (Telerehabilitation* or "tele rehabilitation*" or tele-rehabilitation* or ((remote or virtual) N/1 rehabilitation*))(physical N/1 (exertion or effort)) OR (physical N/2 (endurance or stamina)) OR hydrotherap* OR ((aquatic or "Ai Chi" or pool or water or "water tai chi") N/2 therapy) ) OR AB ( physiotherap* or "physical therap*" OR (Telerehabilitation* or "tele rehabilitation*" or tele-rehabilitation* or ((remote or virtual) N/1 rehabilitation*))(physical N/1 (exertion or effort)) OR (physical N/2 (endurance or stamina)) OR hydrotherap* OR ((aquatic or "Ai Chi" or pool or water or "water tai chi") N/2 therapy) ) ) OR KW ( physiotherap* or "physical therap*" OR (Telerehabilitation* or "tele rehabilitation*" or tele-rehabilitation* or ((remote or virtual) N/1 rehabilitation*))(physical N/1 (exertion or effort)) OR (physical N/2 (endurance or stamina)) OR hydrotherap* OR ((aquatic or "Ai Chi" or pool or water or "water tai chi") N/2 therapy) ) 9,487

S3 ( TI ( (exercise or exercises or aerobic* or "biometric exercise" or "exercise capacity" or "exercise performance" or "exercise training" or "exertion" or "fitness training" or "fitness workout" or "physical conditioning, human" or "physical effort" or "physical exercise" or "physical exertion" or "physical work-out" or "physical workout" or "physical activit*" or Exergam* or (("active video" or active-video) N/2 gaming*) or ("virtual reality" N/1 exercis*) or gymnastics or calisthenics or "physical conditioning" or "physical training" or (("circuit based" or circuit-based or circuit-type) N/1 exercise*) or "circuit training" or "endurance training" or ((high-intensity or "high intensity") N/2 ("interval training*" or "intermittent training*" or "intermittent exercise*")) or "sprint interval training*" or plyometric* or plyometry or ((stretch-shortening or "stretch shortening") N/1 (drill* or exercise* or "cycle exercise*")) or ((pre-operative or preoperative) N/1 (conditioning or exercise* or rehabilitation*)) or prehabilitation or running or jogging or swimming or walking or ambulation or "stair climbing" or "stair navigation" or ((warm-up or warmup or "warm up" or cool-down or cooling-down or "cool* down" or cooldown or "cooling down" or coolingdown or warmdown or warm-down or warming-down or "warming down") N/1 exercise*)) ) OR AB ( (exercise or exercises or aerobic* or "biometric exercise" or "exercise capacity" or "exercise performance" or "exercise training" or "exertion" or "fitness training" or "fitness workout" or "physical conditioning, human" or "physical effort" or "physical exercise" or "physical exertion" or "physical work-out" or "physical workout" or "physical activit*" or Exergam* or (("active video" or active-video) N/2 gaming*) or ("virtual reality" N/1 exercis*) or gymnastics or calisthenics or "physical conditioning" or "physical training" or (("circuit based" or circuit-based or circuit-type) N/1 exercise*) or "circuit training" or "endurance training" or ((high-intensity or "high intensity") N/2 ("interval training*" or "intermittent training*" or "intermittent exercise*")) or "sprint interval training*" or plyometric* or plyometry or ((stretch-shortening or "stretch shortening") N/1 (drill* or exercise* or "cycle exercise*")) or ((pre-operative or preoperative) N/1 (conditioning or exercise* or rehabilitation*)) or prehabilitation or running or jogging or swimming or walking or ambulation or "stair climbing" or "stair navigation" or ((warm-up or warmup or "warm up" or cool-down or cooling-down or "cool* down" or cooldown or "cooling down" or coolingdown or warmdown or warm-down or warming-down or "warming down") N/1 exercise*)) ) ) OR KW ( (exercise or exercises or aerobic* or "biometric exercise" or "exercise capacity" or "exercise performance" or "exercise training" or "exertion" or "fitness training" or "fitness workout" or "physical conditioning, human" or "physical effort" or "physical exercise" or "physical exertion" or "physical work-out" or "physical workout" or "physical activit*" or Exergam* or (("active video" or active-video) N/2 gaming*) or ("virtual reality" N/1 exercis*) or gymnastics or calisthenics or "physical conditioning" or "physical training" or (("circuit based" or circuit-based or circuit-type) N/1 exercise*) or "circuit training" or "endurance training" or ((high-intensity or "high intensity") N/2 ("interval training*" or "intermittent training*" or "intermittent exercise*")) or "sprint interval training*" or plyometric* or plyometry or ((stretch-shortening or "stretch shortening") N/1 (drill* or exercise* or "cycle exercise*")) or ((pre-operative or preoperative) N/1 (conditioning or exercise* or rehabilitation*)) or prehabilitation or running or jogging or swimming or walking or ambulation or "stair climbing" or "stair navigation" or ((warm-up or warmup or "warm up" or cool-down or cooling-down or "cool* down" or cooldown or "cooling down" or coolingdown or warmdown or warm-down or warming-down or "warming down") N/1 exercise*)) ) 147,038

S2 ( (DE "Movement Therapy") OR (DE "Exercise" OR DE "Aerobic Exercise" OR DE "Weightlifting" OR DE "Yoga" OR DE "Sport and Exercise Measures") ) OR ( TI ( kinesiotherap* or kinesitherap* or "corrective exercise*" or "exercise movement technique*" or "exercise therapy" or "exercise treatment*" or "therapeutic exercise" OR ((exercise* N/2 (intervention* or method* or procedure* or technique* or technic* or therap* or treatment*)) or ((bfr or "blood flow restriction*") N/2 (exercise* or therap* or training)) or (endurance N/1 (exercise* or training)) or "closed kinetic chain exercise" or "open kinetic chain exercise*" or "dynamic exercise" or "exercise recovery" or "recovery exercise" or "isokinetic exercise*" or "isometric exercise*" or "isometric endurance" or "isometric training" or "static exercise*" or ((CPM or "continuous passive motion" or "continuous passive movement") N/2 (therapy or therapies)) or "muscle training" or "muscle strengthening" or "myofunctional therapy" or ((active or ballistic or dynamic or isometric or PNF or passive or relaxed or "static active" or static-active or "static passive" or static-passive or static) N/2 (stretching or stretchings)) or "Neuromuscular Facilitation*" or (("cycle exercise*" or drill or drills or exercise*) N/2 (stretch-shortening or "stretch shortening")) or plyometric or plyometrics or "pelvic floor muscle training" or "Kegel exercise*" or "pelvic floor exercise*" or "pelvic floor muscle exercise*" or "pelvic floor training" or "pelvic muscle exercise*" or "pelvic muscle training" or ((resistance or strength) N/2 training) or ((weight-bearing or " weight bearing" or weight-lifting or "weight lifting") N/2 ("exercise program*" or " strengthening program*"))) ) OR AB ( kinesiotherap* or kinesitherap* or "corrective exercise*" or "exercise movement technique*" or "exercise therapy" or "exercise treatment*" or "therapeutic exercise" OR ((exercise* N/2 (intervention* or method* or procedure* or technique* or technic* or therap* or treatment*)) or ((bfr or "blood flow restriction*") N/2 (exercise* or therap* or training)) or (endurance N/1 (exercise* or training)) or "closed kinetic chain exercise" or "open kinetic chain exercise*" or "dynamic exercise" or "exercise recovery" or "recovery exercise" or "isokinetic exercise*" or "isometric exercise*" or "isometric endurance" or "isometric training" or "static exercise*" or ((CPM or "continuous passive motion" or "continuous passive movement") N/2 (therapy or therapies)) or "muscle training" or "muscle strengthening" or "myofunctional therapy" or ((active or ballistic or dynamic or isometric or PNF or passive or relaxed or "static active" or static-active or "static passive" or static-passive or static) N/2 (stretching or stretchings)) or "Neuromuscular Facilitation*" or (("cycle exercise*" or drill or drills or exercise*) N/2 (stretch-shortening or "stretch shortening")) or plyometric or plyometrics or "pelvic floor muscle training" or "Kegel exercise*" or "pelvic floor exercise*" or "pelvic floor muscle exercise*" or "pelvic floor training" or "pelvic muscle exercise*" or "pelvic muscle training" or ((resistance or strength) N/2 training) or ((weight-bearing or " weight bearing" or weight-lifting or "weight lifting") N/2 ("exercise program*" or " strengthening program*"))) OR TI ( ("exercise movement" or "movement therapy" or "motion therapy" or pilates or pilates-based or "breath* exercise*" or "breath* therapy" or "breath* work" or "holotropic breathing" or "Buteyko breathing" or "Buteyko method" or "Buteyko training" or Pranayama or "yoga breathing" or "yogic breathing" or "respiratory muscle training" or Qigong or "ch'i kung" or "qi gong" or (dance N/1 therap*) or "Tai ji" or "t'ai chi" or "tai chi" or "tai ji" or tai-ji or taiji or taijiquan or Yoga) ) OR AB ( ("exercise movement" or "movement therapy" or "motion therapy" or pilates or pilates-based or "breath* exercise*" or "breath* therapy" or "breath* work" or "holotropic breathing" or "Buteyko breathing" or "Buteyko method" or "Buteyko training" or Pranayama or "yoga breathing" or "yogic breathing" or "respiratory muscle training" or Qigong or "ch'i kung" or "qi gong" or (dance N/1 therap*) or "Tai ji" or "t'ai chi" or "tai chi" or "tai ji" or tai-ji or taiji or taijiquan or Yoga) ) ) OR KW ( ( kinesiotherap* or kinesitherap* or "corrective exercise*" or "exercise movement technique*" or "exercise therapy" or "exercise treatment*" or "therapeutic exercise" OR ((exercise* N/2 (intervention* or method* or procedure* or technique* or technic* or therap* or treatment*)) or ((bfr or "blood flow restriction*") N/2 (exercise* or therap* or training)) or (endurance N/1 (exercise* or training)) or "closed kinetic chain exercise" or "open kinetic chain exercise*" or "dynamic exercise" or "exercise recovery" or "recovery exercise" or "isokinetic exercise*" or "isometric exercise*" or "isometric endurance" or "isometric training" or "static exercise*" or ((CPM or "continuous passive motion" or "continuous passive movement") N/2 (therapy or therapies)) or "muscle training" or "muscle strengthening" or "myofunctional therapy" or ((active or ballistic or dynamic or isometric or PNF or passive or relaxed or "static active" or static-active or "static passive" or static-passive or static) N/2 (stretching or stretchings)) or "Neuromuscular Facilitation*" or (("cycle exercise*" or drill or drills or exercise*) N/2 (stretch-shortening or "stretch shortening")) or plyometric or plyometrics or "pelvic floor muscle training" or "Kegel exercise*" or "pelvic floor exercise*" or "pelvic floor muscle exercise*" or "pelvic floor training" or "pelvic muscle exercise*" or "pelvic muscle training" or ((resistance or strength) N/2 training) or ((weight-bearing or " weight bearing" or weight-lifting or "weight lifting") N/2 ("exercise program*" or " strengthening program*"))) ) OR AB ( kinesiotherap* or kinesitherap* or "corrective exercise*" or "exercise movement technique*" or "exercise therapy" or "exercise treatment*" or "therapeutic exercise" OR ((exercise* N/2 (intervention* or method* or procedure* or technique* or technic* or therap* or treatment*)) or ((bfr or "blood flow restriction*") N/2 (exercise* or therap* or training)) or (endurance N/1 (exercise* or training)) or "closed kinetic chain exercise" or "open kinetic chain exercise*" or "dynamic exercise" or "exercise recovery" or "recovery exercise" or "isokinetic exercise*" or "isometric exercise*" or "isometric endurance" or "isometric training" or "static exercise*" or ((CPM or "continuous passive motion" or "continuous passive movement") N/2 (therapy or therapies)) or "muscle training" or "muscle strengthening" or "myofunctional therapy" or ((active or ballistic or dynamic or isometric or PNF or passive or relaxed or "static active" or static-active or "static passive" or static-passive or static) N/2 (stretching or stretchings)) or "Neuromuscular Facilitation*" or (("cycle exercise*" or drill or drills or exercise*) N/2 (stretch-shortening or "stretch shortening")) or plyometric or plyometrics or "pelvic floor muscle training" or "Kegel exercise*" or "pelvic floor exercise*" or "pelvic floor muscle exercise*" or "pelvic floor training" or "pelvic muscle exercise*" or "pelvic muscle training" or ((resistance or strength) N/2 training) or ((weight-bearing or " weight bearing" or weight-lifting or "weight lifting") N/2 ("exercise program*" or " strengthening program*"))) OR ("exercise movement" or "movement therapy" or "motion therapy" or pilates or pilates-based or "breath* exercise*" or "breath* therapy" or "breath* work" or "holotropic breathing" or "Buteyko breathing" or "Buteyko method" or "Buteyko training" or Pranayama or "yoga breathing" or "yogic breathing" or "respiratory muscle training" or Qigong or "ch'i kung" or "qi gong" or (dance N/1 therap*) or "Tai ji" or "t'ai chi" or "tai chi" or "tai ji" or tai-ji or taiji or taijiquan or Yoga) ) 46,154

S1 DE "POSTURAL orthostatic tachycardia syndrome" OR DE "ORTHOSTATIC intolerance" OR TI ( ("orthostatic tachycardia*" or "postural tachycardia*" or "postural tachycardia syndrome" or "postural orthostatic tachycardia syndrome" or ((orthostatic or postural) N/3 (tachycardia* or POTS))) ) OR AB ( ("orthostatic tachycardia*" or "postural tachycardia*" or "postural tachycardia syndrome" or "postural orthostatic tachycardia syndrome" or ((orthostatic or postural) N/3 (tachycardia* or POTS))) ) OR KW ( ("orthostatic tachycardia*" or "postural tachycardia*" or "postural tachycardia syndrome" or "postural orthostatic tachycardia syndrome" or ((orthostatic or postural) N/3 (tachycardia* or POTS))) ) 120

APA PsycInfo legend:

Field codes: TI = title, AB = abstract, KW = keyword, DE = Subject

Proximity operator: N#

Truncation: *

**Psychology and Behavioral Sciences Collection (EBSCOhost)** May 2, 2023

S6 S1 AND (S2 OR S3 OR S4 or S5) **3**

S5 ( (DE "PHYSICAL fitness" OR DE "ANAEROBIC exercises" OR DE "ASTROLOGY & physical fitness" OR DE "BODYBUILDING" OR DE "CARDIOPULMONARY fitness" OR DE "CARDIOVASCULAR fitness" OR DE "CIRCUIT training" OR DE "COMPOUND exercises" OR DE "EXERCISE tolerance" OR DE "ISOLATION exercises" OR DE "LIANGONG" OR DE "MUSCLE strength" OR DE "PERIODIZATION training" OR DE "PHYSICAL fitness for children" OR DE "PHYSICAL fitness for girls" OR DE "PHYSICAL fitness for men" OR DE "PHYSICAL fitness for middle-aged persons" OR DE "PHYSICAL fitness for older people" OR DE "PHYSICAL fitness for people with disabilities" OR DE "PHYSICAL fitness for women" OR DE "PHYSICAL fitness for youth" OR DE "SPORT for all") OR (DE "PHYSICAL training & conditioning" OR DE "ALTITUDE training" OR DE "ANAEROBIC training" OR DE "ARCHERY training" OR DE "BADMINTON training" OR DE "BASE training (Exercise)" OR DE "BASEBALL training" OR DE "BASKETBALL training" OR DE "BICYCLE racing training" OR DE "BODYBUILDING" OR DE "BOWLING training" OR DE "BULLFIGHT training & conditioning" OR DE "CANOEING training" OR DE "CAVING training & conditioning" OR DE "COMPOUND exercises" OR DE "CONTINUOUS training (Exercise)" OR DE "CONTRAST training (Physical training & conditioning)" OR DE "COXSWAINING training" OR DE "CRICKET training & conditioning" OR DE "CROSS-training (Sports)" OR DE "CYCLING training" OR DE "DANCE training & conditioning" OR DE "DEEP diving training & conditioning" OR DE "DIVING training" OR DE "DOGSLEDDING training & conditioning" OR DE "ENDURANCE sports training" OR DE "EXECUTIVES -- Physical training" OR DE "FENCING training" OR DE "FIELD hockey training & conditioning" OR DE "FIREARMS training" OR DE "FOOTBALL training" OR DE "FUNCTIONAL training" OR DE "GLIDING & soaring training & conditioning" OR DE "GOLF training" OR DE "GYMNASTICS training" OR DE "HANDBALL training & conditioning" OR DE "HIKING training & conditioning" OR DE "HOCKEY training" OR DE "HUNTING training & conditioning" OR DE "INTERVAL training" OR DE "ISOLATION exercises" OR DE "KAYAKING training" OR DE "LACROSSE training & conditioning" OR DE "LONG slow distance training" OR DE "MARTIAL arts training" OR DE "MILITARY physical training & conditioning" OR DE "MOTORSPORTS training & conditioning" OR DE "MOUNTAINEERING training" OR DE "ORIENTEERING training" OR DE "OVERTRAINING" OR DE "PACE training" OR DE "PARACHUTING training & conditioning" OR DE "PERIODIZATION training" OR DE "PERSONAL training" OR DE "PHYSICAL training for fire fighters" OR DE "POLICE -- Physical training" OR DE "POLO training & conditioning" OR DE "PRACTICE (Sports)" OR DE "PRESEASON (Sports)" OR DE "RACQUETBALL training" OR DE "RECOVERY training" OR DE "REPETITION training" OR DE "RESISTANCE training" OR DE "ROCK climbing training" OR DE "RODEO training & conditioning" OR DE "ROLLER skating training & conditioning" OR DE "ROWING training" OR DE "RUGBY football training" OR DE "RUNNING training" OR DE "SHOT putting training" OR DE "SKATING training" OR DE "SKI training" OR DE "SKYDIVING training & conditioning" OR DE "SOCCER training" OR DE "SOFTBALL training" OR DE "SPEED endurance training" OR DE "SQUASH training" OR DE "STRENGTH training" OR DE "SURFING training" OR DE "SWIMMING training" OR DE "TABLE tennis training & conditioning" OR DE "TEAM handball training" OR DE "TENNIS training" OR DE "TRACK & field training" OR DE "TRAINING of acrobats" OR DE "TRAINING of boxers (Sports)" OR DE "TRIATHLON training" OR DE "VOLLEYBALL training" OR DE "WATER polo training" OR DE "WEIGHT training" OR DE "WHEELCHAIR sports training" OR DE "WINTER sports training & conditioning" OR DE "WORKING class -- Physical training" OR DE "WRESTLING training" OR DE "YOGA training & conditioning") OR TI ( (physical N/1 (exertion or effort)) OR (physical N/2 (endurance or stamina)) ) OR AB ( (physical N/1 (exertion or effort)) OR (physical N/2 (endurance or stamina)) ) ) OR KW ( (physical N/1 (exertion or effort)) OR (physical N/2 (endurance or stamina)) )

3,551

S4 ( DE "PHYSICAL therapy" OR DE "BALNEOLOGY" OR DE "BATHS" OR DE "BLOOD flow restriction training" OR DE "BODY-weight-supported treadmill training" OR DE "COLD therapy" OR DE "ELECTROTHERAPEUTICS" OR DE "HYDROTHERAPY" OR DE "LIANGONG" OR DE "MANIPULATION therapy" OR DE "NEURODEVELOPMENTAL treatment" OR DE "OCCUPATIONAL therapy" OR DE "PHOTOTHERAPY" OR DE "RECREATIONAL therapy" OR DE "SPORTS physical therapy" OR DE "THERMOTHERAPY" OR DE "VIBRATION therapy" OR DE "TELEREHABILITATION" OR (DE "Telerehabilitation") OR TI ( physiotherap* or "physical therap*" OR (Telerehabilitation* or "tele rehabilitation*" or tele-rehabilitation* or ((remote or virtual) N/1 rehabilitation*))(physical N/1 (exertion or effort)) OR (physical N/2 (endurance or stamina)) OR hydrotherap* OR ((aquatic or "Ai Chi" or pool or water or "water tai chi") N/2 therapy) ) OR AB ( physiotherap* or "physical therap*" OR (Telerehabilitation* or "tele rehabilitation*" or tele-rehabilitation* or ((remote or virtual) N/1 rehabilitation*))(physical N/1 (exertion or effort)) OR (physical N/2 (endurance or stamina)) OR hydrotherap* OR ((aquatic or "Ai Chi" or pool or water or "water tai chi") N/2 therapy) ) ) OR KW ( physiotherap* or "physical therap*" OR (Telerehabilitation* or "tele rehabilitation*" or tele-rehabilitation* or ((remote or virtual) N/1 rehabilitation*))(physical N/1 (exertion or effort)) OR (physical N/2 (endurance or stamina)) OR hydrotherap* OR ((aquatic or "Ai Chi" or pool or water or "water tai chi") N/2 therapy) ) 5,853

S3 ( TI ( (exercise or exercises or aerobic* or "biometric exercise" or "exercise capacity" or "exercise performance" or "exercise training" or "exertion" or "fitness training" or "fitness workout" or "physical conditioning, human" or "physical effort" or "physical exercise" or "physical exertion" or "physical work-out" or "physical workout" or "physical activit*" or Exergam* or (("active video" or active-video) N/2 gaming*) or ("virtual reality" N/1 exercis*) or gymnastics or calisthenics or "physical conditioning" or "physical training" or (("circuit based" or circuit-based or circuit-type) N/1 exercise*) or "circuit training" or "endurance training" or ((high-intensity or "high intensity") N/2 ("interval training*" or "intermittent training*" or "intermittent exercise*")) or "sprint interval training*" or plyometric* or plyometry or ((stretch-shortening or "stretch shortening") N/1 (drill* or exercise* or "cycle exercise*")) or ((pre-operative or preoperative) N/1 (conditioning or exercise* or rehabilitation*)) or prehabilitation or running or jogging or swimming or walking or ambulation or "stair climbing" or "stair navigation" or ((warm-up or warmup or "warm up" or cool-down or cooling-down or "cool* down" or cooldown or "cooling down" or coolingdown or warmdown or warm-down or warming-down or "warming down") N/1 exercise*)) ) OR AB ( (exercise or exercises or aerobic* or "biometric exercise" or "exercise capacity" or "exercise performance" or "exercise training" or "exertion" or "fitness training" or "fitness workout" or "physical conditioning, human" or "physical effort" or "physical exercise" or "physical exertion" or "physical work-out" or "physical workout" or "physical activit*" or Exergam* or (("active video" or active-video) N/2 gaming*) or ("virtual reality" N/1 exercis*) or gymnastics or calisthenics or "physical conditioning" or "physical training" or (("circuit based" or circuit-based or circuit-type) N/1 exercise*) or "circuit training" or "endurance training" or ((high-intensity or "high intensity") N/2 ("interval training*" or "intermittent training*" or "intermittent exercise*")) or "sprint interval training*" or plyometric* or plyometry or ((stretch-shortening or "stretch shortening") N/1 (drill* or exercise* or "cycle exercise*")) or ((pre-operative or preoperative) N/1 (conditioning or exercise* or rehabilitation*)) or prehabilitation or running or jogging or swimming or walking or ambulation or "stair climbing" or "stair navigation" or ((warm-up or warmup or "warm up" or cool-down or cooling-down or "cool* down" or cooldown or "cooling down" or coolingdown or warmdown or warm-down or warming-down or "warming down") N/1 exercise*)) ) ) OR ( (exercise or exercises or aerobic* or "biometric exercise" or "exercise capacity" or "exercise performance" or "exercise training" or "exertion" or "fitness training" or "fitness workout" or "physical conditioning, human" or "physical effort" or "physical exercise" or "physical exertion" or "physical work-out" or "physical workout" or "physical activit*" or Exergam* or (("active video" or active-video) N/2 gaming*) or ("virtual reality" N/1 exercis*) or gymnastics or calisthenics or "physical conditioning" or "physical training" or (("circuit based" or circuit-based or circuit-type) N/1 exercise*) or "circuit training" or "endurance training" or ((high-intensity or "high intensity") N/2 ("interval training*" or "intermittent training*" or "intermittent exercise*")) or "sprint interval training*" or plyometric* or plyometry or ((stretch-shortening or "stretch shortening") N/1 (drill* or exercise* or "cycle exercise*")) or ((pre-operative or preoperative) N/1 (conditioning or exercise* or rehabilitation*)) or prehabilitation or running or jogging or swimming or walking or ambulation or "stair climbing" or "stair navigation" or ((warm-up or warmup or "warm up" or cool-down or cooling-down or "cool* down" or cooldown or "cooling down" or coolingdown or warmdown or warm-down or warming-down or "warming down") N/1 exercise*)) ) 31,539

S2 (DE "MOVEMENT therapy" OR DE "BARTENIEFF Fundamentals (Service mark)" OR DE "CONSTRAINT-induced movement therapy" OR DE "CONTINUUM Movement (Service mark)" OR DE "DANCE therapy" OR DE "DANCE therapy") OR (DE "EXERCISE therapy" OR DE "BLOOD flow restriction training" OR DE "EXERCISE therapy for children" OR DE "EXERCISE therapy for older people" OR DE "SWEDISH gymnastics" OR DE "THERAPEUTIC use of breathing exercises" OR DE "CONTINUOUS passive motion therapy" ) OR (DE "Exercise" OR DE "Aerobic Exercise" OR DE "Weightlifting" OR DE "Yoga" OR DE "Sport and Exercise Measures") ) OR ( TI ( kinesiotherap* or kinesitherap* or "corrective exercise*" or "exercise movement technique*" or "exercise therapy" or "exercise treatment*" or "therapeutic exercise" OR ((exercise* N/2 (intervention* or method* or procedure* or technique* or technic* or therap* or treatment*)) or ((bfr or "blood flow restriction*") N/2 (exercise* or therap* or training)) or (endurance N/1 (exercise* or training)) or "closed kinetic chain exercise" or "open kinetic chain exercise*" or "dynamic exercise" or "exercise recovery" or "recovery exercise" or "isokinetic exercise*" or "isometric exercise*" or "isometric endurance" or "isometric training" or "static exercise*" or ((CPM or "continuous passive motion" or "continuous passive movement") N/2 (therapy or therapies)) or "muscle training" or "muscle strengthening" or "myofunctional therapy" or ((active or ballistic or dynamic or isometric or PNF or passive or relaxed or "static active" or static-active or "static passive" or static-passive or static) N/2 (stretching or stretchings)) or "Neuromuscular Facilitation*" or (("cycle exercise*" or drill or drills or exercise*) N/2 (stretch-shortening or "stretch shortening")) or plyometric or plyometrics or "pelvic floor muscle training" or "Kegel exercise*" or "pelvic floor exercise*" or "pelvic floor muscle exercise*" or "pelvic floor training" or "pelvic muscle exercise*" or "pelvic muscle training" or ((resistance or strength) N/2 training) or ((weight-bearing or " weight bearing" or weight-lifting or "weight lifting") N/2 ("exercise program*" or " strengthening program*"))) ) OR AB ( kinesiotherap* or kinesitherap* or "corrective exercise*" or "exercise movement technique*" or "exercise therapy" or "exercise treatment*" or "therapeutic exercise" OR ((exercise* N/2 (intervention* or method* or procedure* or technique* or technic* or therap* or treatment*)) or ((bfr or "blood flow restriction*") N/2 (exercise* or therap* or training)) or (endurance N/1 (exercise* or training)) or "closed kinetic chain exercise" or "open kinetic chain exercise*" or "dynamic exercise" or "exercise recovery" or "recovery exercise" or "isokinetic exercise*" or "isometric exercise*" or "isometric endurance" or "isometric training" or "static exercise*" or ((CPM or "continuous passive motion" or "continuous passive movement") N/2 (therapy or therapies)) or "muscle training" or "muscle strengthening" or "myofunctional therapy" or ((active or ballistic or dynamic or isometric or PNF or passive or relaxed or "static active" or static-active or "static passive" or static-passive or static) N/2 (stretching or stretchings)) or "Neuromuscular Facilitation*" or (("cycle exercise*" or drill or drills or exercise*) N/2 (stretch-shortening or "stretch shortening")) or plyometric or plyometrics or "pelvic floor muscle training" or "Kegel exercise*" or "pelvic floor exercise*" or "pelvic floor muscle exercise*" or "pelvic floor training" or "pelvic muscle exercise*" or "pelvic muscle training" or ((resistance or strength) N/2 training) or ((weight-bearing or " weight bearing" or weight-lifting or "weight lifting") N/2 ("exercise program*" or " strengthening program*"))) OR TI ( ("exercise movement" or "movement therapy" or "motion therapy" or pilates or pilates-based or "breath* exercise*" or "breath* therapy" or "breath* work" or "holotropic breathing" or "Buteyko breathing" or "Buteyko method" or "Buteyko training" or Pranayama or "yoga breathing" or "yogic breathing" or "respiratory muscle training" or Qigong or "ch'i kung" or "qi gong" or (dance N/1 therap*) or "Tai ji" or "t'ai chi" or "tai chi" or "tai ji" or tai-ji or taiji or taijiquan or Yoga) ) OR AB ( ("exercise movement" or "movement therapy" or "motion therapy" or pilates or pilates-based or "breath* exercise*" or "breath* therapy" or "breath* work" or "holotropic breathing" or "Buteyko breathing" or "Buteyko method" or "Buteyko training" or Pranayama or "yoga breathing" or "yogic breathing" or "respiratory muscle training" or Qigong or "ch'i kung" or "qi gong" or (dance N/1 therap*) or "Tai ji" or "t'ai chi" or "tai chi" or "tai ji" or tai-ji or taiji or taijiquan or Yoga) ) ) OR KW ( ( kinesiotherap* or kinesitherap* or "corrective exercise*" or "exercise movement technique*" or "exercise therapy" or "exercise treatment*" or "therapeutic exercise" OR ((exercise* N/2 (intervention* or method* or procedure* or technique* or technic* or therap* or treatment*)) or ((bfr or "blood flow restriction*") N/2 (exercise* or therap* or training)) or (endurance N/1 (exercise* or training)) or "closed kinetic chain exercise" or "open kinetic chain exercise*" or "dynamic exercise" or "exercise recovery" or "recovery exercise" or "isokinetic exercise*" or "isometric exercise*" or "isometric endurance" or "isometric training" or "static exercise*" or ((CPM or "continuous passive motion" or "continuous passive movement") N/2 (therapy or therapies)) or "muscle training" or "muscle strengthening" or "myofunctional therapy" or ((active or ballistic or dynamic or isometric or PNF or passive or relaxed or "static active" or static-active or "static passive" or static-passive or static) N/2 (stretching or stretchings)) or "Neuromuscular Facilitation*" or (("cycle exercise*" or drill or drills or exercise*) N/2 (stretch-shortening or "stretch shortening")) or plyometric or plyometrics or "pelvic floor muscle training" or "Kegel exercise*" or "pelvic floor exercise*" or "pelvic floor muscle exercise*" or "pelvic floor training" or "pelvic muscle exercise*" or "pelvic muscle training" or ((resistance or strength) N/2 training) or ((weight-bearing or " weight bearing" or weight-lifting or "weight lifting") N/2 ("exercise program*" or " strengthening program*"))) ) OR AB ( kinesiotherap* or kinesitherap* or "corrective exercise*" or "exercise movement technique*" or "exercise therapy" or "exercise treatment*" or "therapeutic exercise" OR ((exercise* N/2 (intervention* or method* or procedure* or technique* or technic* or therap* or treatment*)) or ((bfr or "blood flow restriction*") N/2 (exercise* or therap* or training)) or (endurance N/1 (exercise* or training)) or "closed kinetic chain exercise" or "open kinetic chain exercise*" or "dynamic exercise" or "exercise recovery" or "recovery exercise" or "isokinetic exercise*" or "isometric exercise*" or "isometric endurance" or "isometric training" or "static exercise*" or ((CPM or "continuous passive motion" or "continuous passive movement") N/2 (therapy or therapies)) or "muscle training" or "muscle strengthening" or "myofunctional therapy" or ((active or ballistic or dynamic or isometric or PNF or passive or relaxed or "static active" or static-active or "static passive" or static-passive or static) N/2 (stretching or stretchings)) or "Neuromuscular Facilitation*" or (("cycle exercise*" or drill or drills or exercise*) N/2 (stretch-shortening or "stretch shortening")) or plyometric or plyometrics or "pelvic floor muscle training" or "Kegel exercise*" or "pelvic floor exercise*" or "pelvic floor muscle exercise*" or "pelvic floor training" or "pelvic muscle exercise*" or "pelvic muscle training" or ((resistance or strength) N/2 training) or ((weight-bearing or " weight bearing" or weight-lifting or "weight lifting") N/2 ("exercise program*" or " strengthening program*"))) OR ("exercise movement" or "movement therapy" or "motion therapy" or pilates or pilates-based or "breath* exercise*" or "breath* therapy" or "breath* work" or "holotropic breathing" or "Buteyko breathing" or "Buteyko method" or "Buteyko training" or Pranayama or "yoga breathing" or "yogic breathing" or "respiratory muscle training" or Qigong or "ch'i kung" or "qi gong" or (dance N/1 therap*) or "Tai ji" or "t'ai chi" or "tai chi" or "tai ji" or tai-ji or taiji or taijiquan or Yoga) ) 5,203

S1 DE "POSTURAL orthostatic tachycardia syndrome" OR DE "ORTHOSTATIC intolerance" OR TI ( ("orthostatic tachycardia*" or "postural tachycardia*" or "postural tachycardia syndrome" or "postural orthostatic tachycardia syndrome" or ((orthostatic or postural) N/3 (tachycardia* or POTS))) ) OR AB ( ("orthostatic tachycardia*" or "postural tachycardia*" or "postural tachycardia syndrome" or "postural orthostatic tachycardia syndrome" or ((orthostatic or postural) N/3 (tachycardia* or POTS))) ) OR KW ( ("orthostatic tachycardia*" or "postural tachycardia*" or "postural tachycardia syndrome" or "postural orthostatic tachycardia syndrome" or ((orthostatic or postural) N/3 (tachycardia* or POTS))) ) 54

Psychology and Behavioral Sciences Collection legend:

Field codes: TI = title, AB = abstract, KW = keyword, DE = Subject

Proximity operator: N#

Truncation: *

**SPORTDiscus with Full Text (EBSCOhost)** May 2, 2023

S6 S1 AND (S2 OR S3 OR S4 OR S5) **28**

S5 ( (DE "PHYSICAL fitness testing" OR DE "EUROFIT" OR DE "HEART function tests" OR DE "JUMP & reach tests" OR DE "KRAUS-Weber test" OR DE "MUSCLE strength measurement" OR DE "PHYSICAL Activity Readiness Questionnaire" OR DE "PHYSICAL fitness testing for children" OR DE "STEP tests") OR (DE "PHYSICAL fitness" OR DE "ANAEROBIC exercises" OR DE "ASTROLOGY & physical fitness" OR DE "BODYBUILDING" OR DE "CARDIOPULMONARY fitness" OR DE "CARDIOVASCULAR fitness" OR DE "CIRCUIT training" OR DE "COMPOUND exercises" OR DE "EXERCISE tolerance" OR DE "ISOLATION exercises" OR DE "LIANGONG" OR DE "MUSCLE strength" OR DE "PERIODIZATION training" OR DE "PHYSICAL fitness for children" OR DE "PHYSICAL fitness for girls" OR DE "PHYSICAL fitness for men" OR DE "PHYSICAL fitness for older people" OR DE "PHYSICAL fitness for people with disabilities" OR DE "PHYSICAL fitness for women" OR DE "PHYSICAL fitness for youth" OR DE "SPORT for all") ) OR ( OR TI ( (physical N/1 (exertion or effort)) OR (physical N/2 (endurance or fitness or stamina)) ) OR AB ( (physical N/1 (exertion or effort)) OR (physical N/2 (endurance or fitness or stamina)) ) ) OR KW ( (physical N/1 (exertion or effort)) OR (physical N/2 (endurance or fitness or stamina)) ) 121,740

S4 ( (DE "PHYSICAL therapy" OR DE "BALNEOLOGY" OR DE "COLD therapy" OR DE "ELECTROTHERAPEUTICS" OR DE "HYDROTHERAPY" OR DE "LIANGONG" OR DE "MANIPULATION therapy" OR DE "OCCUPATIONAL therapy" OR DE "PHOTOTHERAPY" OR DE "RECREATIONAL therapy" OR DE "SPORTS physical therapy" OR DE "THERMOTHERAPY" ) ) OR ( OR TI ( physiotherap* or "physical therap*" OR (Telerehabilitation* or "tele rehabilitation*" or tele-rehabilitation* or ((remote or virtual) N/1 rehabilitation*))(physical N/1 (exertion or effort)) OR (physical N/2 (endurance or stamina)) OR hydrotherap* OR ((aquatic or "Ai Chi" or pool or water or "water tai chi") N/2 therapy) ) OR AB ( physiotherap* or "physical therap*" OR (Telerehabilitation* or "tele rehabilitation*" or tele-rehabilitation* or ((remote or virtual) N/1 rehabilitation*))(physical N/1 (exertion or effort)) OR (physical N/2 (endurance or stamina)) OR hydrotherap* OR ((aquatic or "Ai Chi" or pool or water or "water tai chi") N/2 therapy) ) ) OR KW ( physiotherap* or "physical therap*" OR (Telerehabilitation* or "tele rehabilitation*" or tele-rehabilitation* or ((remote or virtual) N/1 rehabilitation*))(physical N/1 (exertion or effort)) OR (physical N/2 (endurance or stamina)) OR hydrotherap* OR ((aquatic or "Ai Chi" or pool or water or "water tai chi") N/2 therapy) ) ) 39,127

S3 ( (DE "EXERCISE" OR DE "ABDOMINAL exercises" OR DE "AEROBIC exercises" OR DE "ANAEROBIC exercises" OR DE "AQUATIC exercises" OR DE "ARM exercises" OR DE "BACK exercises" OR DE "BREATHING exercises" OR DE "BREEMA" OR DE "BUTTOCKS exercises" OR DE "CALISTHENICS" OR DE "CHAIR exercises" OR DE "CHEST exercises" OR DE "CIRCUIT training" OR DE "COMPOUND exercises" OR DE "COOLDOWN" OR DE "DO-in" OR DE "EXERCISE adherence" OR DE "EXERCISE for children" OR DE "EXERCISE for girls" OR DE "EXERCISE for men" OR DE "EXERCISE for middle-aged persons" OR DE "EXERCISE for older people" OR DE "EXERCISE for people with disabilities" OR DE "EXERCISE for women" OR DE "EXERCISE for youth" OR DE "EXERCISE therapy" OR DE "EXERCISE video games" OR DE "FACIAL exercises" OR DE "FALUN gong exercises" OR DE "FOOT exercises" OR DE "GYMNASTICS" OR DE "HAND exercises" OR DE "HATHA yoga" OR DE "HIP exercises" OR DE "ISOKINETIC exercise" OR DE "ISOLATION exercises" OR DE "ISOMETRIC exercise" OR DE "ISOTONIC exercise" OR DE "KNEE exercises" OR DE "LEG exercises" OR DE "LIANGONG" OR DE "METABOLIC equivalent" OR DE "MULAN quan" OR DE "MUSCLE strength" OR DE "PILATES method" OR DE "PLYOMETRICS" OR DE "QI gong" OR DE "REDUCING exercises" OR DE "RUNNING" OR DE "SCHOOL exercises & recreations" OR DE "SEXUAL exercises" OR DE "SHOULDER exercises" OR DE "STRENGTH training" OR DE "STRESS management exercises" OR DE "TAI chi" OR DE "TREADMILL exercise" OR DE "WHEELCHAIR workouts" OR DE "YOGA") ) OR ( ( TI ( (exercise or exercises or aerobic* or "biometric exercise" or "exercise capacity" or "exercise performance" or "exercise training" or "exertion" or "fitness training" or "fitness workout" or "physical conditioning, human" or "physical effort" or "physical exercise" or "physical exertion" or "physical work-out" or "physical workout" or "physical activit*" or Exergam* or (("active video" or active-video) N/2 gaming*) or ("virtual reality" N/1 exercis*) or gymnastics or calisthenics or "physical conditioning" or "physical training" or (("circuit based" or circuit-based or circuit-type) N/1 exercise*) or "circuit training" or "endurance training" or ((high-intensity or "high intensity") N/2 ("interval training*" or "intermittent training*" or "intermittent exercise*")) or "sprint interval training*" or plyometric* or plyometry or ((stretch-shortening or "stretch shortening") N/1 (drill* or exercise* or "cycle exercise*")) or ((pre-operative or preoperative) N/1 (conditioning or exercise* or rehabilitation*)) or prehabilitation or running or jogging or swimming or walking or ambulation or "stair climbing" or "stair navigation" or ((warm-up or warmup or "warm up" or cool-down or cooling-down or "cool* down" or cooldown or "cooling down" or coolingdown or warmdown or warm-down or warming-down or "warming down") N/1 exercise*)) ) OR AB ( (exercise or exercises or aerobic* or "biometric exercise" or "exercise capacity" or "exercise performance" or "exercise training" or "exertion" or "fitness training" or "fitness workout" or "physical conditioning, human" or "physical effort" or "physical exercise" or "physical exertion" or "physical work-out" or "physical workout" or "physical activit*" or Exergam* or (("active video" or active-video) N/2 gaming*) or ("virtual reality" N/1 exercis*) or gymnastics or calisthenics or "physical conditioning" or "physical training" or (("circuit based" or circuit-based or circuit-type) N/1 exercise*) or "circuit training" or "endurance training" or ((high-intensity or "high intensity") N/2 ("interval training*" or "intermittent training*" or "intermittent exercise*")) or "sprint interval training*" or plyometric* or plyometry or ((stretch-shortening or "stretch shortening") N/1 (drill* or exercise* or "cycle exercise*")) or ((pre-operative or preoperative) N/1 (conditioning or exercise* or rehabilitation*)) or prehabilitation or running or jogging or swimming or walking or ambulation or "stair climbing" or "stair navigation" or ((warm-up or warmup or "warm up" or cool-down or cooling-down or "cool* down" or cooldown or "cooling down" or coolingdown or warmdown or warm-down or warming-down or "warming down") N/1 exercise*)) ) ) OR ( (exercise or exercises or aerobic* or "biometric exercise" or "exercise capacity" or "exercise performance" or "exercise training" or "exertion" or "fitness training" or "fitness workout" or "physical conditioning, human" or "physical effort" or "physical exercise" or "physical exertion" or "physical work-out" or "physical workout" or "physical activit*" or Exergam* or (("active video" or active-video) N/2 gaming*) or ("virtual reality" N/1 exercis*) or gymnastics or calisthenics or "physical conditioning" or "physical training" or (("circuit based" or circuit-based or circuit-type) N/1 exercise*) or "circuit training" or "endurance training" or ((high-intensity or "high intensity") N/2 ("interval training*" or "intermittent training*" or "intermittent exercise*")) or "sprint interval training*" or plyometric* or plyometry or ((stretch-shortening or "stretch shortening") N/1 (drill* or exercise* or "cycle exercise*")) or ((pre-operative or preoperative) N/1 (conditioning or exercise* or rehabilitation*)) or prehabilitation or running or jogging or swimming or walking or ambulation or "stair climbing" or "stair navigation" or ((warm-up or warmup or "warm up" or cool-down or cooling-down or "cool* down" or cooldown or "cooling down" or coolingdown or warmdown or warm-down or warming-down or "warming down") N/1 exercise*)) ) ) 504,509

S2 (DE "MOVEMENT therapy" OR DE "CONSTRAINT-induced movement therapy" OR DE "DANCE therapy") OR (DE "EXERCISE therapy" OR DE "EXERCISE therapy for children" OR DE "EXERCISE therapy for older people" OR DE "MENSENDIECK system" OR DE "ORTHOPTICS" OR DE "SWEDISH gymnastics" OR DE "THERAPEUTIC use of breathing exercises" ) OR OR ( TI ( kinesiotherap* or kinesitherap* or "corrective exercise*" or "exercise movement technique*" or "exercise therapy" or "exercise treatment*" or "therapeutic exercise" OR ((exercise* N/2 (intervention* or method* or procedure* or technique* or technic* or therap* or treatment*)) or ((bfr or "blood flow restriction*") N/2 (exercise* or therap* or training)) or (endurance N/1 (exercise* or training)) or "closed kinetic chain exercise" or "open kinetic chain exercise*" or "dynamic exercise" or "exercise recovery" or "recovery exercise" or "isokinetic exercise*" or "isometric exercise*" or "isometric endurance" or "isometric training" or "static exercise*" or ((CPM or "continuous passive motion" or "continuous passive movement") N/2 (therapy or therapies)) or "muscle training" or "muscle strengthening" or "myofunctional therapy" or ((active or ballistic or dynamic or isometric or PNF or passive or relaxed or "static active" or static-active or "static passive" or static-passive or static) N/2 (stretching or stretchings)) or "Neuromuscular Facilitation*" or (("cycle exercise*" or drill or drills or exercise*) N/2 (stretch-shortening or "stretch shortening")) or plyometric or plyometrics or "pelvic floor muscle training" or "Kegel exercise*" or "pelvic floor exercise*" or "pelvic floor muscle exercise*" or "pelvic floor training" or "pelvic muscle exercise*" or "pelvic muscle training" or ((resistance or strength) N/2 training) or ((weight-bearing or " weight bearing" or weight-lifting or "weight lifting") N/2 ("exercise program*" or " strengthening program*"))) ) OR AB ( kinesiotherap* or kinesitherap* or "corrective exercise*" or "exercise movement technique*" or "exercise therapy" or "exercise treatment*" or "therapeutic exercise" OR ((exercise* N/2 (intervention* or method* or procedure* or technique* or technic* or therap* or treatment*)) or ((bfr or "blood flow restriction*") N/2 (exercise* or therap* or training)) or (endurance N/1 (exercise* or training)) or "closed kinetic chain exercise" or "open kinetic chain exercise*" or "dynamic exercise" or "exercise recovery" or "recovery exercise" or "isokinetic exercise*" or "isometric exercise*" or "isometric endurance" or "isometric training" or "static exercise*" or ((CPM or "continuous passive motion" or "continuous passive movement") N/2 (therapy or therapies)) or "muscle training" or "muscle strengthening" or "myofunctional therapy" or ((active or ballistic or dynamic or isometric or PNF or passive or relaxed or "static active" or static-active or "static passive" or static-passive or static) N/2 (stretching or stretchings)) or "Neuromuscular Facilitation*" or (("cycle exercise*" or drill or drills or exercise*) N/2 (stretch-shortening or "stretch shortening")) or plyometric or plyometrics or "pelvic floor muscle training" or "Kegel exercise*" or "pelvic floor exercise*" or "pelvic floor muscle exercise*" or "pelvic floor training" or "pelvic muscle exercise*" or "pelvic muscle training" or ((resistance or strength) N/2 training) or ((weight-bearing or " weight bearing" or weight-lifting or "weight lifting") N/2 ("exercise program*" or " strengthening program*"))) OR TI ( ("exercise movement" or "movement therapy" or "motion therapy" or pilates or pilates-based or "breath* exercise*" or "breath* therapy" or "breath* work" or "holotropic breathing" or "Buteyko breathing" or "Buteyko method" or "Buteyko training" or Pranayama or "yoga breathing" or "yogic breathing" or "respiratory muscle training" or Qigong or "ch'i kung" or "qi gong" or (dance N/1 therap*) or "Tai ji" or "t'ai chi" or "tai chi" or "tai ji" or tai-ji or taiji or taijiquan or Yoga) ) OR AB ( ("exercise movement" or "movement therapy" or "motion therapy" or pilates or pilates-based or "breath* exercise*" or "breath* therapy" or "breath* work" or "holotropic breathing" or "Buteyko breathing" or "Buteyko method" or "Buteyko training" or Pranayama or "yoga breathing" or "yogic breathing" or "respiratory muscle training" or Qigong or "ch'i kung" or "qi gong" or (dance N/1 therap*) or "Tai ji" or "t'ai chi" or "tai chi" or "tai ji" or tai-ji or taiji or taijiquan or Yoga) ) ) OR KW ( ( kinesiotherap* or kinesitherap* or "corrective exercise*" or "exercise movement technique*" or "exercise therapy" or "exercise treatment*" or "therapeutic exercise" OR ((exercise* N/2 (intervention* or method* or procedure* or technique* or technic* or therap* or treatment*)) or ((bfr or "blood flow restriction*") N/2 (exercise* or therap* or training)) or (endurance N/1 (exercise* or training)) or "closed kinetic chain exercise" or "open kinetic chain exercise*" or "dynamic exercise" or "exercise recovery" or "recovery exercise" or "isokinetic exercise*" or "isometric exercise*" or "isometric endurance" or "isometric training" or "static exercise*" or ((CPM or "continuous passive motion" or "continuous passive movement") N/2 (therapy or therapies)) or "muscle training" or "muscle strengthening" or "myofunctional therapy" or ((active or ballistic or dynamic or isometric or PNF or passive or relaxed or "static active" or static-active or "static passive" or static-passive or static) N/2 (stretching or stretchings)) or "Neuromuscular Facilitation*" or (("cycle exercise*" or drill or drills or exercise*) N/2 (stretch-shortening or "stretch shortening")) or plyometric or plyometrics or "pelvic floor muscle training" or "Kegel exercise*" or "pelvic floor exercise*" or "pelvic floor muscle exercise*" or "pelvic floor training" or "pelvic muscle exercise*" or "pelvic muscle training" or ((resistance or strength) N/2 training) or ((weight-bearing or " weight bearing" or weight-lifting or "weight lifting") N/2 ("exercise program*" or " strengthening program*"))) ) OR AB ( kinesiotherap* or kinesitherap* or "corrective exercise*" or "exercise movement technique*" or "exercise therapy" or "exercise treatment*" or "therapeutic exercise" OR ((exercise* N/2 (intervention* or method* or procedure* or technique* or technic* or therap* or treatment*)) or ((bfr or "blood flow restriction*") N/2 (exercise* or therap* or training)) or (endurance N/1 (exercise* or training)) or "closed kinetic chain exercise" or "open kinetic chain exercise*" or "dynamic exercise" or "exercise recovery" or "recovery exercise" or "isokinetic exercise*" or "isometric exercise*" or "isometric endurance" or "isometric training" or "static exercise*" or ((CPM or "continuous passive motion" or "continuous passive movement") N/2 (therapy or therapies)) or "muscle training" or "muscle strengthening" or "myofunctional therapy" or ((active or ballistic or dynamic or isometric or PNF or passive or relaxed or "static active" or static-active or "static passive" or static-passive or static) N/2 (stretching or stretchings)) or "Neuromuscular Facilitation*" or (("cycle exercise*" or drill or drills or exercise*) N/2 (stretch-shortening or "stretch shortening")) or plyometric or plyometrics or "pelvic floor muscle training" or "Kegel exercise*" or "pelvic floor exercise*" or "pelvic floor muscle exercise*" or "pelvic floor training" or "pelvic muscle exercise*" or "pelvic muscle training" or ((resistance or strength) N/2 training) or ((weight-bearing or " weight bearing" or weight-lifting or "weight lifting") N/2 ("exercise program*" or " strengthening program*"))) OR ("exercise movement" or "movement therapy" or "motion therapy" or pilates or pilates-based or "breath* exercise*" or "breath* therapy" or "breath* work" or "holotropic breathing" or "Buteyko breathing" or "Buteyko method" or "Buteyko training" or Pranayama or "yoga breathing" or "yogic breathing" or "respiratory muscle training" or Qigong or "ch'i kung" or "qi gong" or (dance N/1 therap*) or "Tai ji" or "t'ai chi" or "tai chi" or "tai ji" or tai-ji or taiji or taijiquan or Yoga) ) 22,957

S1 DE "ORTHOSTATIC intolerance" OR TI ( ("orthostatic tachycardia*" or "postural tachycardia*" or "postural tachycardia syndrome" or "postural orthostatic tachycardia syndrome" or ((orthostatic or postural) N/3 (tachycardia* or POTS))) ) OR AB ( ("orthostatic tachycardia*" or "postural tachycardia*" or "postural tachycardia syndrome" or "postural orthostatic tachycardia syndrome" or ((orthostatic or postural) N/3 (tachycardia* or POTS))) ) OR KW ( ("orthostatic tachycardia*" or "postural tachycardia*" or "postural tachycardia syndrome" or "postural orthostatic tachycardia syndrome" or ((orthostatic or postural) N/3 (tachycardia* or POTS))) ) **98**

SportDiscus with Full Text legend:

Field codes: TI = title, AB = abstract, KW = keyword, DE = Subject

Proximity operator: N#

Truncation: *

**Scopus (Elsevier, scopus.com)** May 2, 2023

( TITLE-ABS-KEY ( ( "orthostatic tachycardia*" OR "postural tachycardia*" OR "postural tachycardia syndrome" OR "postural orthostatic tachycardia syndrome" OR ( ( orthostatic OR postural ) W/3 ( tachycardia* OR pots ) ) ) OR ( "orthostatic intolerance" OR "orthostatic dysregulation" ) ) ) AND ( ( TITLE-ABS-KEY ( ( kinesiotherap* OR kinesitherap* OR "corrective exercise*" OR "exercise movement technique*" OR "exercise therapy" OR "exercise treatment*" OR "therapeutic exercise" ) OR ( ( exercise* W/2 ( intervention* OR method* OR procedure* OR technique* OR technic* OR therap* OR treatment* ) ) OR ( ( bfr OR "blood flow restriction*" ) W/2 ( exercise* OR therap* OR training ) ) OR ( endurance W/1 ( exercise* OR training ) ) OR "closed kinetic chain exercise" OR "open kinetic chain exercise*" OR "dynamic exercise" OR "exercise recovery" OR "recovery exercise" OR "isokinetic exercise*" OR "isometric exercise*" OR "isometric endurance" OR "isometric training" OR "static exercise*" OR ( ( cpm OR "continuous passive motion" OR "continuous passive movement" ) W/2 ( therapy OR therapies ) ) OR "muscle training" OR "muscle strengthening" OR "myofunctional therapy" OR ( ( active OR ballistic OR dynamic OR isometric OR pnf OR passive OR relaxed OR "static active" OR static-active OR "static passive" OR static-passive OR static ) W/2 ( stretching OR stretchings ) ) OR "Neuromuscular Facilitation*" OR ( ( "cycle exercise*" OR drill OR drills OR exercise* ) W/2 ( stretch-shortening OR "stretch shortening" ) ) OR plyometric OR plyometrics OR "pelvic floor muscle training" OR "Kegel exercise*" OR "pelvic floor exercise*" OR "pelvic floor muscle exercise*" OR "pelvic floor training" OR "pelvic muscle exercise*" OR "pelvic muscle training" OR ( ( resistance OR strength ) W/2 training ) OR ( ( weight-bearing OR " weight bearing" OR weight-lifting OR "weight lifting" ) W/2 ( "exercise program*" OR " strengthening program*" ) ) ) OR ( ( aquatic OR "Ai Chi" OR pool OR water OR "water tai chi" ) W/2 therapy ) ) OR hydrotherap* ) OR ( TITLE-ABS-KEY ( ( "exercise movement" OR "movement therapy" OR "motion therapy" OR pilates OR pilates-based OR "breath* exercise*" OR "breath* therapy" OR "breath* work" OR "holotropic breathing" OR "Buteyko breathing" OR "Buteyko method" OR "Buteyko training" OR pranayama OR "yoga breathing" OR "yogic breathing" OR "respiratory muscle training" OR qigong OR "ch'i kung" OR "qi gong" OR ( dance W/1 therap* ) OR "Tai ji" OR "t'ai chi" OR "tai chi" OR "tai ji" OR tai-ji OR taiji OR taijiquan OR yoga ) OR ( exercise OR exercises OR aerobic* OR "biometric exercise" OR "exercise capacity" OR "exercise performance" OR "exercise training" OR "exertion" OR "fitness training" OR "fitness workout" OR "physical conditioning, human" OR "physical effort" OR "physical exercise" OR "physical exertion" OR "physical work-out" OR "physical workout" OR "physical activit*" OR exergam* OR ( ( "active video" OR active-video ) W/2 gaming* ) OR ( "virtual reality" W/1 exercis* ) OR gymnastics OR calisthenics OR "physical conditioning" OR "physical training" OR ( ( "circuit based" OR circuit-based OR circuit-type ) W/1 exercise* ) OR "circuit training" OR "endurance training" OR ( ( high-intensity OR "high intensity" ) W/2 ( "interval training*" OR "intermittent training*" OR "intermittent exercise*" ) ) OR "sprint interval training*" OR plyometric* OR plyometry OR ( ( stretch-shortening OR "stretch shortening" ) W/1 ( drill* OR exercise* OR "cycle exercise*" ) ) OR ( ( pre-operative OR preoperative ) W/1 ( conditioning OR exercise* OR rehabilitation* ) ) OR prehabilitation OR running OR jogging OR swimming OR walking OR ambulation OR "stair climbing" OR "stair navigation" OR ( ( warm-up OR warmup OR "warm up" OR cool-down OR cooling-down OR "cool* down" OR cooldown OR "cooling down" OR coolingdown OR warmdown OR warm-down OR warming-down OR "warming down" ) W/1 exercise* ) ) OR ( physiotherap* OR "physical therap*" ) OR ( telerehabilitation* OR "tele rehabilitation*" OR tele-rehabilitation* OR ( ( remote OR virtual ) W/1 rehabilitation* ) ) ) ) OR ( TITLE-ABS-KEY ( ( physical W/1 ( exertion OR effort ) ) OR ( physical W/2 ( endurance OR fitness OR stamina ) ) ) ) ) AND ( EXCLUDE ( DOCTYPE , "cr" ) ) **853** results

Scopus legend:

Field codes: TITLE-ABS-KEY = Document Title, Abstract, Keyword

Proximity operator: W/

Truncation: *

**Web of Science Core Collection (Clarivate)** May 2, 2023

# Entitlements:

- Science Citation Index Expanded (SCI-EXPANDED) --1900-present
- Social Sciences Citation Index (SSCI) --1900-present
- Arts & Humanities Citation Index (A&HCI) --1975-present
- Emerging Sources Citation Index (ESCI) --2018-present

# Searches:

1: TS=( ("orthostatic tachycardia*" or "postural tachycardia*" or "postural tachycardia syndrome"

or "postural orthostatic tachycardia syndrome" or ((orthostatic or postural) NEAR/3 (tachycardia*

or POTS))) OR "orthostatic intolerance" or "orthostatic dysregulation") Results: 3385

2: TS=( kinesiotherap* or kinesitherap* or "corrective exercise*" or "exercise movement

technique*" or "exercise therapy" or "exercise treatment*" or "therapeutic exercise" OR

((exercise* NEAR/2 (intervention* or method* or procedure* or technique* or technic* or therap*

or treatment*)) or ((bfr or "blood flow restriction*") NEAR/2 (exercise* or therap* or training)) or

(endurance NEAR/1 (exercise* or training)) or "closed kinetic chain exercise" or "open kinetic

chain exercise*" or "dynamic exercise" or "exercise recovery" or "recovery exercise" or

"isokinetic exercise*" or "isometric exercise*" or "isometric endurance" or "isometric training" or

"static exercise*" or ((CPM or "continuous passive motion" or "continuous passive movement")

NEAR/2 (therapy or therapies)) or "muscle training" or "muscle strengthening" or "myofunctional

therapy" or ((active or ballistic or dynamic or isometric or PNF or passive or relaxed or "static

active" or static-active or "static passive" or static-passive or static) NEAR/2 (stretching or

stretchings)) or "Neuromuscular Facilitation*" or (("cycle exercise*" or drill or drills or exercise*)

NEAR/2 (stretch-shortening or "stretch shortening")) or plyometric or plyometrics or "pelvic floor

muscle training" or "Kegel exercise*" or "pelvic floor exercise*" or "pelvic floor muscle exercise*"

or "pelvic floor training" or "pelvic muscle exercise*" or "pelvic muscle training" or ((resistance or

strength) NEAR/2 training) or ((weight-bearing or " weight bearing" or weight-lifting or "weight

lifting") NEAR/2 ("exercise program*" or " strengthening program*"))) OR ((aquatic or "Ai Chi" or

pool or water or "water tai chi") NEAR/2 therapy) OR ("exercise movement" or "movement

therapy" or "motion therapy" or pilates or pilates-based or "breath* exercise*" or "breath*

therapy" or "breath* work" or "holotropic breathing" or "Buteyko breathing" or "Buteyko method"

or "Buteyko training" or Pranayama or "yoga breathing" or "yogic breathing" or "respiratory

muscle training" or Qigong or "ch'i kung" or "qi gong" or (dance NEAR/1 therap*) or "Tai ji" or "t'ai

chi" or "tai chi" or "tai ji" or tai-ji or taiji or taijiquan or Yoga) ) Results: 112455

3: TS=( (exercise or exercises or aerobic* or "biometric exercise" or "exercise capacity" or

"exercise performance" or "exercise training" or "exertion" or "fitness training" or "fitness

workout" or "physical conditioning, human" or "physical effort" or "physical exercise" or "physical

exertion" or "physical work-out" or "physical workout" or "physical activit*" or Exergam* or

(("active video" or active-video) NEAR/2 gaming*) or ("virtual reality" NEAR/1 exercis*) or

gymnastics or calisthenics or "physical conditioning" or "physical training" or (("circuit based" or

circuit-based or circuit-type) NEAR/1 exercise*) or "circuit training" or "endurance training" or

((high-intensity or "high intensity") NEAR/2 ("interval training*" or "intermittent training*" or

"intermittent exercise*")) or "sprint interval training*" or plyometric* or plyometry or

((stretch-shortening or "stretch shortening") NEAR/1 (drill* or exercise* or "cycle exercise*")) or

((pre-operative or preoperative) NEAR/1 (conditioning or exercise* or rehabilitation*)) or

prehabilitation or running or jogging or swimming or walking or ambulation or "stair climbing" or

"stair navigation" or ((warm-up or warmup or "warm up" or cool-down or cooling-down or "cool*

down" or cooldown or "cooling down" or coolingdown or warmdown or warm-down or

warming-down or "warming down") NEAR/1 exercise*)) OR physiotherap* or "physical therap*"

OR yoga OR (Telerehabilitation* or "tele rehabilitation*" or tele-rehabilitation* or ((remote or

virtual) NEAR/1 rehabilitation*)) OR (physical NEAR/1 (exertion or effort)) OR (physical NEAR/2

(endurance or stamina))) Results: 1555315

4: #2 OR #3 Results: 1572526

5: #1 AND #4 Results: 603

6: #1 AND #4 and Meeting Abstract (Exclude – Document Types) Results: **586**

Web of Science Core Collection legend:

Field codes: TS = topic

Proximity operator: NEAR/#

Truncation: *
