# Supplementary material for: Impact of exercise to treat postural orthostatic tachycardia syndrome: a systematic review
Source: Front Neurol. 2025 Apr 24;16:1567708. doi: 10.3389/fneur.2025.1567708 (PMC12071195; doi:10.3389/fneur.2025.1567708)
Supplement: Supplementary file 2 [file Table_2.docx]

**Supplement 2**

**Included Studies**

1a. Fu Q, Vangundy TB, Galbreath MM, et al. Cardiac origins of the postural orthostatic tachycardia syndrome. *J Am Coll Cardiol*. 2010;55(25):2858-68. doi:10.1016/j.jacc.2010.02.043

1b. Fu Q, Vangundy TB, Shibata S, Auchus RJ, Williams GH, Levine BD. Exercise training versus propranolol in the treatment of the postural orthostatic tachycardia syndrome. *Hypertension*. 2011;58(2):167-75. doi:10.1161/HYPERTENSIONAHA.111.172262

2. Galbreath MM, Shibata S, VanGundy TB, Okazaki K, Fu Q, Levine BD. Effects of exercise training on arterial-cardiac baroreflex function in POTS. *Clin Auton Res*. 2011;21(2):73-80. doi:10.1007/s10286-010-0091-5

3. George SA, Bivens TB, Howden EJ, et al. The international POTS registry: Evaluating the efficacy of an exercise training intervention in a community setting. *Heart Rhythm*. 2016;13(4):943-50. doi:10.1016/j.hrthm.2015.12.012

4. Gibbons CH, Silva G, Freeman R. Cardiovascular exercise as a treatment of postural orthostatic tachycardia syndrome: A pragmatic treatment trial. *Heart Rhythm*. 2021;18(8):1361-1368. doi:10.1016/j.hrthm.2021.01.017

5. Shibata S, Fu Q, Bivens TB, Hastings JL, Wang W, Levine BD. Short-term exercise training improves the cardiovascular response to exercise in the postural orthostatic tachycardia syndrome. *J Physiol*. 2012;590(15):3495-505. doi:10.1113/jphysiol.2012.233858

6. Wheatley-Guy CM, Shea MG, Parks JK, et al. Semi-supervised exercise training program more effective for individuals with postural orthostatic tachycardia syndrome in randomized controlled trial. *Clin Auton Res*. Dec 2023;33(6):659-672. doi:10.1007/s10286-023-00970-w

7. Winker R, Barth A, Bidmon D, et al. Endurance exercise training in orthostatic intolerance: a randomized, controlled trial. *Hypertension*. Mar 2005;45(3):391-8. doi:10.1161/01.HYP.0000156540.25707.af
