# Supplementary material for: Impact of exercise to treat postural orthostatic tachycardia syndrome: a systematic review
Source: Front Neurol. 2025 Apr 24;16:1567708. doi: 10.3389/fneur.2025.1567708 (PMC12071195; doi:10.3389/fneur.2025.1567708)
Supplement: Supplementary file 3 [file Table_3.docx]

**Supplement 3**

**Excluded Studies with reasons for exclusion**

1. Effect of Exercise in OI. Trial registry record; Clinical trial protocol. Source: https://clinicaltrials.gov/show/NCT00770484. 2008. Cochrane Central Register of Controlled Trials (CENTRAL) [CN-01597786]

Exclusion reason: Clinical trial registration, incomplete/unpublished.

2. Assessing the feasibility of a supervised exercise rehabilitation intervention with behavioural and motivational support, for people with postural orthostatic tachycardia syndrome. Trial registry record; Clinical trial protocol. Source: https://trialsearch.who.int/Trial2.aspx?TrialID=ISRCTN45323485. 2020. Cochrane Central Register of Controlled Trials (CENTRAL) [CN-02172233]

Exclusion reason: Clinical trial registration, incomplete/unpublished.

3. Remote Self-training Program for Patients With Postural Orthostatic Tachycardia Syndrome. Trial registry record; Clinical trial protocol. Source: https://clinicaltrials.gov/show/NCT04603157. 2020. Cochrane Central Register of Controlled Trials (CENTRAL) [CN-02183932]

Exclusion reason: Clinical trial registration, incomplete/unpublished.

4. Breathing Exercises With And Without Aerobic Training In Patients With Postural Orthostatic Tachycardia Syndrome. Trial registry record; Clinical trial protocol. Source: https://clinicaltrials.gov/show/NCT05404672. 2022. Cochrane Central Register of Controlled Trials (CENTRAL) [CN-02405690]

Exclusion reason: Clinical trial registration, incomplete/unpublished.

5. The Effect of Physical Activity on Postural Orthostatic Tachycardia Syndrome. Trial registry record; Clinical trial protocol. Source: https://clinicaltrials.gov/show/NCT05554107. 2022. Cochrane Central Register of Controlled Trials (CENTRAL) [CN-02467052]

Exclusion reason: Clinical trial registration, incomplete/unpublished.

6. Physiology of Long COVID-19 and the Impact of Cardiopulmonary Rehabilitation on Quality-of-Life and Functional Capacity. Trial registry record; Clinical trial protocol. Source: https://clinicaltrials.gov/show/NCT05566483. 2022. Cochrane Central Register of Controlled Trials (CENTRAL) [CN-02475159]

Exclusion reason: Clinical trial registration, incomplete/unpublished.

7. Aliyev F. Postural orthostatic tachycardia: A syndrome requiring detailed search for associated conditions. *Turk Kardiyoloji Dernegi Arsivi*. 2017;45(3):214-216. doi:10.5543/tkda.2017.77943.

Exclusion reason: Wrong study design.

8. Armstrong KR, Souza AM, Sneddon PL, Potts JE, Claydon VE, Sanatani S. Exercise and the multidisciplinary holistic approach to adolescent dysautonomia. *Acta Paediatr*. 2017;106(4):612-618. doi:10.1111/apa.13750.

Exclusion reason: Wrong patient population, data unavailable.

9. Benito KG, Ramanathan A, Lobato D, et al. Symptoms, impairment and treatment needs among youth with orthostatic intolerance in a secondary care setting. *Child Health Care*. 2022;51(3):316-335. doi:10.1080/02739615.2022.2047049.

Exclusion reason: Wrong intervention.

10. Bruce BK, Harrison TE, Bee SM, et al. Improvement in Functioning and Psychological Distress in Adolescents With Postural Orthostatic Tachycardia Syndrome Following Interdisciplinary Treatment. *Clin Pediatr (Phila)*. 2016;55(14):1300-1304. doi:10.1177/0009922816638663.

Exclusion reason: Wrong outcomes. interventions used.

11. Crawford MH. Exercise Training for POTS. *Clinical Cardiology Alert*. 2010;29(8):63-63.

Exclusion reason: Conference presentation.

12. Cui Y, Liao Y, Zhang Q, et al. Spectrum of underlying diseases in syncope and treatment of neurally-mediated syncope in children and adolescents over the past 30 years: A single center study. *Frontiers in Cardiovascular Medicine*. 2022;9:1017505. doi:10.3389/fcvm.2022.1017505.

Exclusion reason: Wrong intervention.

13. Cui YX, Du JB, Zhang QY, et al. A 10-year retrospective analysis of spectrums and treatment options of orthostatic intolerance and sitting intolerance in children. *Beijing da Xue Xue Bao Yi Xue Ban/Journal of Peking University Health Sciences*. 2022;54(5):954-960.

Exclusion reason: Non-English. No translation funding available.

14. Fu Q, Levine BD. Exercise in the postural orthostatic tachycardia syndrome. *Autonomic Neuroscience-Basic & Clinical*. 2015;188:86-9. doi:10.1016/j.autneu.2014.11.008.

Exclusion reason: Wrong study design.

15. Gauthier N, Reynolds L, Curran T, O'Neill J, Gauvreau K, Alexander ME. FORCE Risk Stratification Tool for Pediatric Cardiac Rehabilitation and Fitness Programs. *Pediatr Cardiol*. 2022;19:19. doi:10.1007/s00246-022-03010-y.

Exclusion reason: Wrong outcomes.

16. Hainsworth R. Exercise training in orthostatic intolerance. *QJM*. 1998;91(11):715-7.

Exclusion reason: Wrong study design.

17. Hutt E, Vajapey R, Van Iterson EH, et al. Functional capacity and quality of life in the postural tachycardia syndrome: A retrospective cross-sectional study. *Annals of Medicine & Surgery*. 2020;56:72-76. doi:10.1016/j.amsu.2020.06.013.

Exclusion reason: Wrong intervention.

18. Joyner MJ. Exercise training in Postural Orthostatic Tachycardia syndrome: blocking the urge to block beta-receptors? *Hypertension*. 2011;58(2):136-7. doi:10.1161/HYPERTENSIONAHA.111.173872.

Exclusion reason: Wrong study design.

19. Joyner MJ. Exercise training in postural orthostatic tachycardia syndrome: Blocking the urge to block β-receptors? *Hypertension*. 2011;58(2):136-137. doi:10.1161/HYPERTENSIONAHA.111.173872.

Exclusion reason: Wrong study design.

20. Klaas KM, Fischer PR, Segner S, et al. Excessive Postural Tachycardia and Postural Orthostatic Tachycardia Syndrome in Youth: Associations With Distress, Impairment, Health Behaviors, and Medication Recommendations. *J Child Neurol*. 2022;37(7):599-608. doi:10.1177/08830738221078410.

Exclusion reason: Wrong intervention.

21. Lu W, Yan H, Wu S, et al. Electrocardiography-Derived Predictors for Therapeutic Response to Treatment in Children with Postural Tachycardia Syndrome. *J Pediatr*. 2016;176:128-33. doi:10.1016/j.jpeds.2016.05.030.

Exclusion reason: Wrong intervention.

22. McGregor G, Evans B, Sandhu H, et al. Protocol update for a randomised controlled feasibility trial of exercise rehabilitation for people with postural tachycardia syndrome: the PULSE study. *Pilot & Feasibility Studies*. 2022;8(1):101. doi:10.1186/s40814-022-01056-6.

Exclusion reason: Clinical trial registration, incomplete/unpublished.

23. McGregor G, Hee SW, Eftekhari H, et al. Protocol for a randomised controlled feasibility trial of exercise rehabilitation for people with postural tachycardia syndrome: the PULSE study. *Pilot & Feasibility Studies*. 2020;6:157. doi:10.1186/s40814-020-00702-1.

Exclusion reason: Clinical trial registration, incomplete/unpublished.

24. Miyazaki H, Kurihara W, Han X, Sakaguchi S, Kushiyama K, Spencer SN. Development of Exergame to Resolve Deconditioning in Children with Orthostatic Dysregulation. *Proceedings - SIGGRAPH 2022 Posters*. 2022;doi:10.1145/3532719.3543212.

Exclusion reason: Wrong patient population.

25. Pianosi PT, Goodloe AH, Soma D, Parker KO, Brands CK, Fischer PR. High flow variant postural orthostatic tachycardia syndrome amplifies the cardiac output response to exercise in adolescents. *Physiological Reports*. 2014;2(8):01. doi:10.14814/phy2.12122.

Exclusion reason: Wrong study design.

26. Pianosi PT, Schroeder DR, Fischer PR. Cardiac responses to exercise distinguish postural orthostatic tachycardia syndrome variants. *Physiological Reports*. 2016;4(22):11.

Exclusion reason: Wrong intervention.

27. Privett SE, George KP, Whyte GP, Cable NT. The effectiveness of compression garments and lower limb exercise on post-exercise blood pressure regulation in orthostatically intolerant athletes. *Clin J Sport Med*. 2010;20(5):362-7. doi:10.1097/JSM.0b013e3181f20292.

Exclusion reason: Wrong intervention.

28. Reilly CC, Floyd SV, Lee K, et al. Breathlessness and dysfunctional breathing in patients with postural orthostatic tachycardia syndrome (POTS): The impact of a physiotherapy intervention. *Autonomic Neuroscience-Basic & Clinical*. 2020;223:102601. doi:10.1016/j.autneu.2019.102601.

Exclusion reason: Wrong intervention.

29. Rudofker EW, Parker H, Cornwell WK, 3rd. An Exercise Prescription as a Novel Management Strategy for Treatment of Long COVID. *JACC Case Reports*. 2022;4(20):1344-1347. doi:10.1016/j.jaccas.2022.06.026.

Exclusion reason: Wrong study design.

30. Sutcliffe K, Gray J, Tan MP, et al. Home orthostatic training in chronic fatigue syndrome - A randomized, placebo-controlled feasibility study. *Eur J Clin Invest*. 2010;40(1):18-24. doi:10.1111/j.1365-2362.2009.02225.x.

Exclusion reason: Wrong patient population.

31. Uechi T, Suda T, Hirabuki K, et al. Effectiveness of Three-Dimensional Echocardiography for Asian Postural Orthostatic Tachycardia Syndrome during Exercise Therapy: 845 Board #106 May 30 3:30 PM - 5:00 PM...ACSM 2018, American College of Sports Medicine Annual Meeting, May 29-June 2, 2018, Minneapolis, MN, USA. *Med Sci Sports Exerc*. 2018;50:190-190. doi:10.1249/01.mss.0000535710.45061.f6.

Exclusion reason: Wrong study design.

32. Usmani S, Anum S, Patwary M, et al. Tachycardia Induced Cardiomyopathy (TIC) in Patients with Postural Orthostatic Tachycardia Syndrome (POTS) and Ehlers-Danlos Syndromes (EDS). *J Card Fail*. 2019;25(8):S86. doi:10.1016/j.cardfail.2019.07.245.

Exclusion reason: Conference presentation.

33. Van Iterson EH, Laffin LJ, Mayuga KA, et al. High Submaximal Exercise Heart Rate Impacts Exercise Intolerance in the Postural Orthostatic Tachycardia Syndrome. *J Cardiopulm Rehabil Prev*. 2020;40(3):195-201. doi:10.1097/HCR.0000000000000485.

Exclusion reason: Wrong intervention..

34. Wheatley-Guy CM, Shea MG, Parks JK, Scales R, Goodman BP, Johnson BD. Semi-supervised exercise training program more efficacious for individuals with postural orthostatic tachycardia syndrome. *Clin Auton Res*. 2022;32(5):379. doi:10.1007/s10286-022-00892-z.

Exclusion reason: Conference presentation.

35. Wheatley-Guy CM, Shea MG, Parks JK, Scales R, Johson BD. Efficacy Of A Semi-supervised Exercise Training Program For Individuals With Postural Orthostatic Tachycardia Syndrome: 1224...American College of Sports Medicine, Annual Meeting and World Congresses, May 31-June 4, 2022, San Diego, California. *Med Sci Sports Exerc*. 2022;54(9S):300-300. doi:10.1249/01.mss.0000878788.01507.c6.

Exclusion reason: Conference presentation.

36. Wheatley-Guy CM, Shea MG, Parks JK, Scales R, Johson BD. Efficacy Of A Semi-supervised Exercise Training Program For Individuals With Postural Orthostatic Tachycardia Syndrome: 1224. *Med Sci Sports Exerc*. 2022;54(9S):300-300.

Exclusion reason: Conference presentation.
